# Interactive effects of seed rate, sowing method, and variety on Teff (*Eragrostis tef*) yield, yield components, and lodging trait performance in the central highlands of Ethiopia

**DOI:** 10.1371/journal.pone.0360297

**Published:** 2026-10-12

**Authors:** Yemanebirhane Tesfa, Fresew Belete, Wondwosen Tena

**Affiliations:** 1 Department of Plant Sciences, College of Agriculture and Natural Sciences, Debre Berhan University, Debre Berhan, Ethiopia; 2 Department of Plant Biology and Biodiversity Management, College of Natural and Computational Sciences, Addis Ababa University, Addis Ababa, Ethiopia; KGUT: Graduate University of Advanced Technology, IRAN, ISLAMIC REPUBLIC OF

## Abstract

Teff (Eragrostis tef) occupies close to three million hectares of agricultural land in Ethiopia, nonetheless, realized yields remain substantially below the crop’s inherent genetic yield potential. Thus, this study aimed to examine how different sowing rates and methods impacted productivity of teff varieties in Mojana Wodera Woreda. The treatments included three varieties of teff (namely, *Quncho*, *Kora* and *Abolse* (local variety), three sowing rates (10, 15 and 20 kg ha ^−1^), and three sowing methods (15 cm and 20 cm spacing between rows and broad casting). The interaction effect of seed rate, sowing method and variety significantly affected crop growth and yield parameters (P < 0.01). The tallest plant (128.33 cm) was achieved from variety *Quncho* combined with 20 cm row spacing and at a seed rate of 10 kg ha^–1^. *Quncho* sown at a rate of 10 kg ha^–1^ with a row spacing of 15 cm produced the maximum amount of aboveground biomass yield (10.76 t ha^–1^). Furthermore, the highest lodging index (44%) was recorded when variety *Quncho* was sown with a broadcasting method at the highest seed rate of 20 kg ha^–1^. The main effect of seed rate, sowing method and variety significantly affected days to 50% heading (P < 0.05), panicle length and grain yield (P < 0.01). Variety *Kora* significantly took longer time (59.18) to reach 50% heading than variety *Quncho* and *Abolse*. Increasing row spacing to 20 cm prolong the time for 50% heading of teff varieties, while sowing teff variety at a a rate of 15 kg ha^–1^ also significantly increased the days to heading. *Quncho* produced the largest panicle length (45.14 cm) and yield (2.47 t ha^–1^) which is 15.96% higher than variety *Kora* and 26.67% higher than the grain yield obtained from the local *Abolse* variety. Furthermore, sowing teff at a row spacing of 20 cm produced the maximum panicle length (44.46 cm) and yield (2.32 t ha^–1^). As to the seed rate, thhe highest grain yield and largest panicle were when teff was seeded at 10 kg ha^–1^.

## Introduction

Teff (*Eragrostis tef* [Zucc.] Trotter) is an ancient, nutrient-dense cereal grain native to the Horn of Africa [1]. Globally, it has gained significant international prominence as a “superfood” and premium gluten-free alternative, driving expanded commercial cultivation in countries such as the United States, Australia, South Africa, and parts of Europe [2,3]. Despite its expanding global footprint, the African continent remains the epicenter of teff production, accounting for over 95% of world output [4]. Within Africa, Ethiopia is the dominant primary producer, contributing more than 90% of global teff production across an estimated 3 million hectares [5,6]. The crop plays an indispensable role in regional food security and rural livelihoods, serving as a primary dietary staple for millions while offering resilience to diverse agro-ecological conditions [7].

Teff (*Eragrostis tef*) is a highly valued crop for its robust ecological tolerance and its foundational importance to the Ethiopia’s cultural nutrition. It is an annual, self-pollinated, allotetraploid (20 chromosome pairs) [8], C4 crop belonging to the Poaceae family. The crop is domesticated and diverse in Ethiopia [9]. Its value is the largest in the country in terms of both production and consumption [6,10]. Teff is predominantly used to prepare injera, a traditional spongy flatbread that constitutes the main dietary staple in Ethiopia. Therefore, it can be considered an alternative to gluten-containing cereals such as wheat, barley and rye. Furthermore, teff is known for rich iron content [11], and the people consuming it as their main staple food are found in Ethiopia, where it plays a vital role in national food security and human nutrition [11]. Teff grows at altitudes of 1700–2800 m, faces few pests or diseases [12] and tolerates both drought and waterlogging, making it a low-risk, resilient crop [6].

In Ethiopia, Teff ranks first in area coverage but has the lowest productivity. It accounts for 23.85% of the land allotted for cereals and 17.26% of the grain produced from cereals, with meager national average productivity of 1.76 t ha^–1^ [5]. In Amhara region, teff ranks first in area coverage, with more than one million hectares, and is the most significant cereal cultivated by the majority of farmers [5]. With respect to the study area (Mojana Wodera), the North Showa Zone, teff covers more than 186 thousand hectares, and it accounts for nearly 40.7% of the total allocated for cereal crops in the 2016 main cropping season [5] Despite its importance, its productivity in the country, particularly in the Amhara region’s Mojana Wodera Woreda, faces substantial production challenges that hinder its yield potential. The major challenges include lodging, the use of traditional sowing methods, improper seed rates, and the use of low yielding local varieties [13].

The seed size of teff is very small, and it creates a havoc during sowing, as it is difficult to control the distribution and population density. Farmers sow the crop at a higher seed rate than the recommended rate, mainly because of the use of unclean seeds with poor germination rates, which results in a 22% reduction in both straw and grain yield [14]. The seed rate and sowing method are essential agronomic aspects that demand careful consideration. The seed rate significantly influences crop growth and development by determining plant density. Traditionally, teff is sown via the broadcasting method at 25−30 kg ha^–1^. This resulted in a suboptimal plant population per area, which increased nutrient, water, sunlight, and CO_2_ competition. Additionally, compared with unit row spacing, broadcasting increases production costs because it requires a higher seed rate. Broadcasting also causes lodging, which is the main reason for the low yield of teff [13]. Lodging issues in teff can be minimized by using a lower seeding rate, planting in rows and selecting the proper variety [15]. Row planting in teff has a greater yield advantage compared to broadcasting [16]. Various studies have suggested different row spacings for teff. A spacing of 20 cm and 15 cm between rows was suggested by [17] and [18], respectively.

Previous agronomic studies in Ethiopia have evaluated individual components of teff productivity, yet notable geographic and methodology; specific gaps remain. For instance, studies conducted at the Debre Zeit and Holetta Agricultural Research Centers demonstrated that row sowing generally improves grain yield over traditional broadcasting by enhancing light interception and reducing seed rate requirements [19,20]. Similarly, evaluation trials conducted in the moist mid-highlands of the Amhara Region (such as Adet and Debre Tabor) identified significant yield variations among improved varieties like *Kora* and Dagim when subjected to varying nitrogen rates and plant densities [21].

However, a critical limitation in existing literature is that most prior studies examined seed rates, sowing methods, or varieties in isolation or dual-factorial arrangements, leaving a gap in understanding their three-way interactive effects (seed rate, sowing method and variety). Furthermore, previous findings from centralized research stations cannot be directly extrapolated to specific localized agro-ecologies like Mojana Wodera Woreda, where microclimatic variations particularly seasonal distribution of rainfall and ambient temperatures differ markedly from regional trial centers. Evaluating these three factors simultaneously in Mojana Wodera, Zubaba keble under seasonal rainfall and temperature is essential to generate reliable, site-specific agronomic recommendations for local smallholders.

In the Amhara region, particularly in Mojana Wodera Woreda, farmers use broadcasting for sowing. However, farmers harness low yields due to suboptimal agronomic practices, including a lack of variety specific guidelines for row spacing and seeding [13]. This research investigates how seeding rate and sowing method influence the productivity of different teff genotypes.

## Materials and Methods

### Study area

The field experiment was conducted during 2022 main cropping season (Meher) in Mojana Wodera woreda at Zubaba Keble (70°55’ N and 39°37’ E) in central Ethiopia. No specific permits or ethical approvals were required for this study, as the work was conducted on publicly accessible land and did not involve protected species, human participants, or regulated animal subjects. The woreda receives 852 mm of rainfall annually and is known for producing rain-fed teff in Ethiopia (Fig 1). The average daily temperatures of the woreda ranges between 12.5°C and 20.5°C. The soil type is mainly pure vertisol, which occupy approximately 4045 ha.

**Fig 1:**
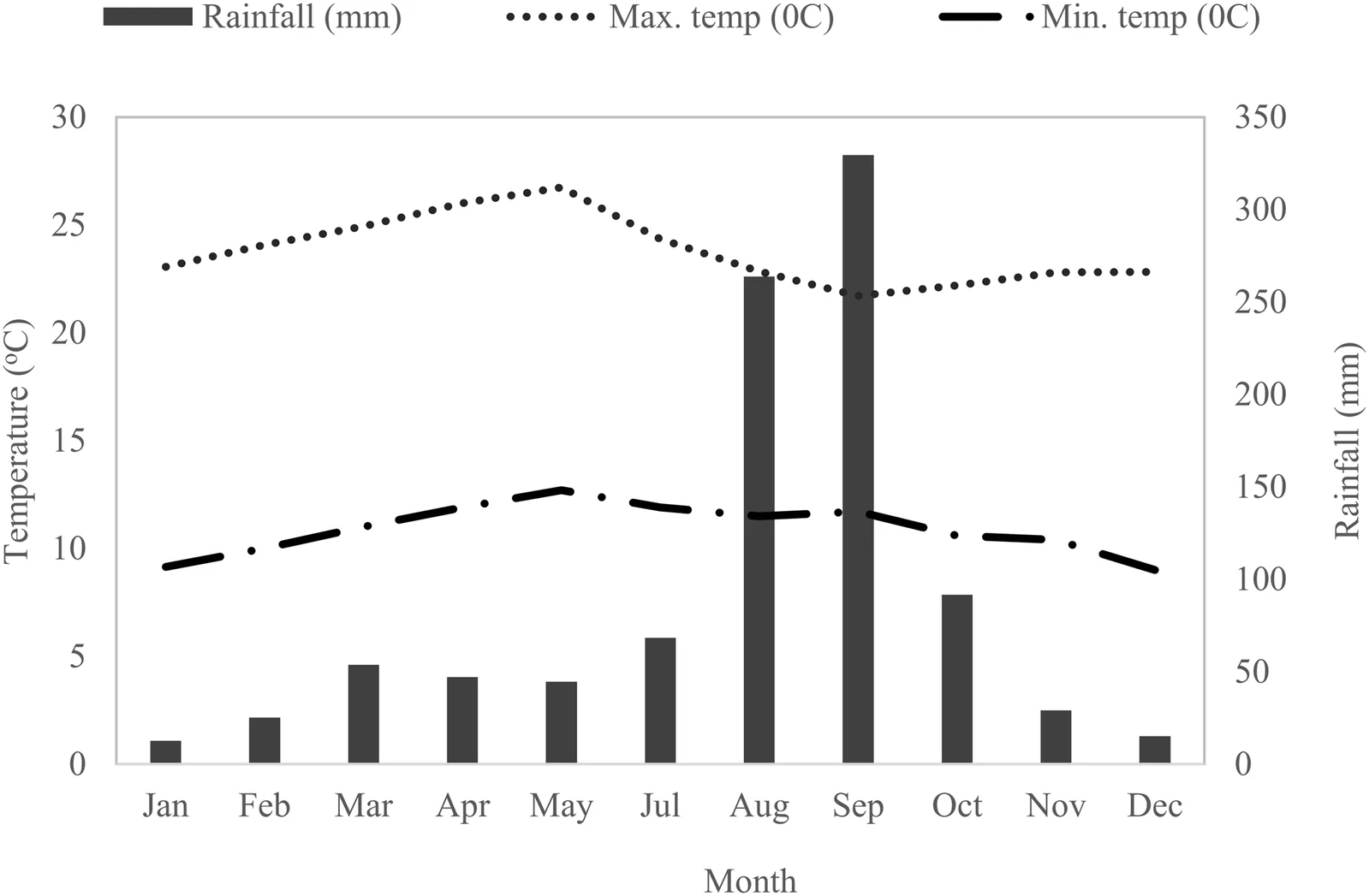
Rainfall, minimum and maximum temperatures during 2022 in Mojana wodera woreda.

### Experimental design and cultural practice

Three distinct teff genotypes: *Abolse* (a local landrace), *kora* and *Quncho* were evaluated using three seed rates (10, 15 and 20 kg ha^–1^) and three methods of sowing. Seeds of these varieties were officially obtained from the Debre Zeit Agricultural Research Center of the Ethiopian Institute of Agricultural Research Center, which serves as the national center of excellence for teff improvement in Ethiopia. The three methods of sowing comprised of a traditional broadcast method alongside tow row planting at row spacing of 0.15m and 0.2m, respectively. Factorial randomized block design with three replications was used. Each experimental plot was 3 × 1.5 m^2^. For row spacing treatment each plot contained 18 rows spaced at 15 cm. A blended Nitrogen, Phosphorus, Sulphur and Boron (NPSB) fertilizer at a recommended rate (100 kg ha^–1^) was applied at sowing, while 100 kg ha^–1^ urea was applied, with half of it applied 15–18 days after seeding and the rest applied at the crop tillering stage [22]. The plots were kept weed free. No insecticides or fungicides were used, as there were no insect or disease outbreaks.

### Data collection

The parameters measured included the days to plant height, (PH)50% heading (D50H), number of tillers per plant (NT), panicle length (PL), biomass yield (BY), and grain yield (GY). Lodging index (LI): the extent of lodging was assessed visually by observing the angle between the main stem’s inclination and the vertical base. A scale of 1–5 was used: 1 (0–15°) indicates no lodging, 2 (15--30°) indicates 25% lodging, 3 (30–45°) indicates 50% lodging, 4 (45–60°) indicates 75% lodging, and 5 (60–90°) indicates 100% lodging [23].

### Statistical analysis

A three-way ANOVA was performed on the dataset to determine if there were variations among the teff genotypes in response to sowing rate and sowing method utilizing R statistical package Version R-4.05. Mean comparison was performed via the least significance difference (LSD) test at the 5% level of significance.

## Results and discussions

### Plant height

Plant height is a crucial growth attribute that influences the productivity of a crop, affecting both yield and straw. It is also positively correlated with increased production and productivity of crops [24]. The mean square analysis in this study revealed a significant difference in the height of teff due to the main effects of variety, sowing method, and seeding rate (Table 1). Among varieties, variety *Quncho* produced the tallest plants (114.6 cm), exceeding the local variety *Kora* and *Abolse* by 10.05% and 25.99%, respectively (Table 3). For sowing method row sowing of teff with 20 cm recorded the higher mean plant height (108.17 cm) than broadcasting (99.14 cm) (Table 3). As to seed rate, the lowest rate (10 kg ha^–1^) resulted in the highest plant height (107.97 cm). However, because the three-way interaction effect among factors was significant (P < 0.01), the biological response is primarily governed by their specific combination. Variety *Quncho* reached the tallest height (128.33 cm) when sown at 20 cm row spacing and a 10 kg ha^–1^ seed rate. However, the local *Abolse* cultivar produced the shortest value (84.83 cm) when the seed rate was doubled to 20 kg ha^–1^ and rows were narrowed to 15 cm (Table 2). The interaction effect of the *Quncho* variety, sown at 10 kg ha^–1^ with 20 cm between rows, resulted in a 43.5 cm increase in height compared with the local *Abolse* variety seeded at 20 kg ha^–1^ and 15 cm between rows (Table 2).

**Table 1:** Mean squares from the ANOVA for phenological, growth, and yield related traits of teff varieties as affected by the effects of the seed rate and sowing method in the Mojana Wodera woreda.

Source of variation	Parameters						
	PH	DH	NT	PL	BY	GY	LI
Rep (2)	5.07	26.43	0.53	11.34	0.31	0.21	1.51
V (2)	3791**	18.06*	11.26**	211.35**	13.48**	0.1.89**	417.51**
SR (2)	646.37**	19.69*	5.52**	32.92**	11.04**	0.35*	1071.73**
SM (2)	562.94**	19.25*	6.12**	76.35**	10.32**	0.49**	288.86**
SR*SM (4)	122.07**	1.59NS	1.34*	1.06NS	0.89**	0.06NS	11.72**
V*SR (4)	19.38*	0.44NS	0.58NS	0.83NS	0.57**	0.06NS	10.85**
V*SM (4)	56.9***	3.22NS	0.02NS	0.71NS	0.39***	0.03NS	9.4***
V*SR*SM (8)	44.17**	0.24NS	0.15NS	0.55NS	0.4***	0.01NS	5.61**
Error	5.84	1.18	0.34	2.18	0.06	0.04	0.96

Where Rep = replication; V = variety; SR = seed rate; and SM = sowing method, NS = non-significant ** = highly significant, numbers in bracket indicate the degree of freedom. PH (cm) = Plant height, DT = Days to heading, NT = Number of tillers per plant, PL (cm)= Panicle length, BY (t ha^-1^) = biomass yield, GY (t ha^-1^) = Grain yield, LI (%) = Lodging index.

**Table 2:** Interaction effects of variety, sowing method, and seed rate on plant height (cm) of teff in Mojana Wodera Woreda.

		Seed rate (kg ha^–1^)		
**Variety**	**Sowing method (cm)**	**10**	**15**	**20**
*Quncho*	15	122^b^	116 cd	110.8^e^
	20	128.33^a^	117.67^c^	114.2^cde^
	BC	112^e^	112^e^	98.77^hi^
*Kora*	15	111.2^e^	94.17^j^	99.5^ghi^
	20	111.1^e^	112.4^de^	106.13^f^
	BC	100.97^gh^	105.867^f^	96.17^ji^
*Abolse*	15	95.93ij	88^k^	84.83^k^
	20	103.33^gf^	93.47^j^	86.9^k^
	BC	86.9^k^	93^j^	86.57^k^
LSD (5%)	3.95			
CV (%)	2.34			

Mean values sharing a common letter are non-significant (P > 0.05) based on least significance Difference (LSD). CV denotes coefficient of variation. BC = Broadcasting

In general, plant height significantly increased as the seeding rates were lowered from 20 kg ha^–1^ to 10 kg ha^–1^ during broadcast sowing, as the additional space between plants allowed for better growth. These findings suggest that using lower seed rates with the drilling method resulted in taller teff plants. This is corroborated by [25], who reported 8.1 cm height advantage when using 5 kg ha^-1^ rate versus 25 kg ha^-1^ seeding rate. Wider row spacing at constant seed rates tended to produce taller plants [26].

The variation in teff stature among different cultivars might also be attributed to their distinct genomic compositions. A similar result was reported by [13], where compared with the other tested varieties, *Quncho* had the tallest plant height (130.94 cm). However [27], reported a different result where the local variety surpassed the height of *Quncho*, which exhibited a comparatively shorter stature in that specific environment.

### Days to 50% heading (DH)

The mean square analysis of variance confirmed that variety, row spacing, and seed rate independently impacted days to 50% heading significantly, their combined interactions (two-way and three-way) remained non-significant (Table 1). Significantly, a greater number of days to heading (59.36 and 58.37 days) were recorded from the lower seeding rates (10 and 15 kg ha^–1^), whereas the minimum number of days (57.66 days) was from the highest seeding rate (20 kg ha^–1^) (Table 3). The earlier heading under higher seeding rates might be induced as a competitive stress response, causing the teff crop to enter the reproductive phase to ensure seed production before resources are exhausted. On the contrary, a lower seed rate reduces competition and enables plants to access necessary growth resources, which leads to vegetative growth extension, resulting in a delay in the heading of the teff crop. An increase in planting density hastens the inflorescence emergence in rice plants [16]. In contrast to the findings of this study [25], reported a significant ontogenetic delay in teff when it was sown at 20 kg ha ^−1^.

**Table 3:** Impact of different variety, sowing methods and seed rates on plant height (PH), days to heading (DH), number of tillers per plant (NT), biomass yield (BY) and lodging index (LI) of teff in Mojana Wodera Woreda.

Variety	PH (cm)	DH	NT	BY (t ha^–1^)	LI (%)
*Quncho*	114.64^a^	58.63^a^	6.4^a^	8.65^a^	38.18^a^
*Kora*	104.17^b^	59.18^a^	5.68^b^	7.71^b^	30.33^c^
*Abolse*	90.99^c^	57.57^b^	5.11^c^	7.27^c^	34.65^b^
**Sowing method (cm)**					
15	102.49^b^	58.40^b^	5.66^b^	8.32^a^	27.18c
20	108.17^a^	59.33^a^	6.24^a^	8.14^b^	37.11b
BC	99.14^c^	57.65^b^	5.29^c^	7.17^c^	38.87 a
**Seed rate (kg ha**^–**1**^)					
10	107.97^a^	59.36^a^	6.19^a^	8.46^a^	27.18^c^
15	103.62^b^	58.37^b^	5.71^b^	7.99^b^	37.11^b^
20	98.21^c^	57.66^b^	5.29^c^	7.19^c^	38.87^a^
LSD (0.05)	1.32	0.59	0.32		0.54
CV (%)	2.34	1.85	10.13	3.13	2.85

Mean values sharing a common letter are non-significant (P > 0.05) based on least significance Difference (LSD). CV denotes coefficient of variation. BC = broadcasting.

Among the varieties, *Kora* took a significantly longer time (59.18 days) to heading than did the variety *Quncho* (58.63 days) and the local variety *Abolse* (57.57 days) (Table 3). The variation in heading date could be due to the genetic differences among the three cultivars in response to different seeding rates and planting methods. Similarly, the time required for teff to reach heading stage varies by cultivar.

With respect to the sowing method, the present study revealed that sowing teff with 20 cm row spacing significantly delays the number of days to heading compared with that with narrower 15 cm spacing and broadcasting (Table 3). The wider spacing and improved management within rows may have reduced intraspecific competition, allowing efficient utilization of growth resources. The earlier heading of teff plants observed in this study with narrow spacing may be due to the increased competition for resources in closer rows. This competition might have caused the plants to experience resource shortages, leading them to head earlier to escape stressful conditions rather than extending their vegetative growth and biomass accumulation. Additionally, genetic factors may play a role [13].

### Number of tillers

The mean square analysis demonstrated that tiller production per plant was significantly (P < 0.01) influenced by variety, sowing method and seed rate (Table 1). Variety *Quncho* produced the highest number of tiller (6.4 tillers/plant), exceeding variety *Kora* (5.68 tillers/plant) by 12.68% and the local variety (5.11 tilers/plant) by 25.24%. As to sowing method, row sowing of teff with 20 cm spacing increased tiller production (6.24 tillers/plant) over traditional broadcasting (5.29 tillers/plat) by 17.96%. For seed rate, lower seeding rate (10 kg ha^–1^) resulted in higher tiller counts (6.19 tillers/plant), recording a 17.01% increase over higher seeding rate (20 kg ha^–1^, 5.29 tillers/plant) (Table 3). Furthermore, sowing methods and seed rate interactions also had notable effects on this trait. But neither the two-way interaction effect of varieties with sowing method and varieties with seed rate nor by three-way interaction effect of varieties, sowing method and seed rate were significant (Table 1). The highest tillering capacity (7.09 per plant) was achieved through a seeding rate of 10 kg ha^–1^ combined with 20 cm row spacing. In contrast, broadcasting yielded to a significantly the lowest tiller count per plant (Table 4). This finding highlights a low seed rate and increased inter-row spacing optimize plant access to nutrients and light allowing plants to express their full tillering potential, while denser populations lead to nutrient stress that stunts tillering [28]. These result aligns with the findings of [26], who noted that lowering teff seeding rate from 20 to10 kg ha^–1^ during broadcast conditions enhanced tiller production by alleviating spatial constraints. Similarly [29], reported that increasing the seeding density reduced total and productive tillers in teff crops. Compared to broadcasting method, the row sowing result garnered in significantly more tillers per plant [9]. In contrast [30], confirmed that seeding rate had no significant effect on individual plant tillering, while broadcasting at different seed rates significantly influenced the total tiller populations compared to structured row arrangement.

**Table 4:** Tillering performance of teff as a function of row spacing and seed rate in Mojana Wodera Woreda district.

Sowing method (cm)	Seed rate kg ha^–1^		
	10	15	20
15	6.2^b^	5.56 cd	5.22^de^
20	7.09^a^	5.95^bc^	5.67^bcd^
BC	5.29^de^	5.61 cd	4.98^e^
CV%	10.13		
LSD (0.05)	0.55		

Mean values sharing a common letter are non-significant (P > 0.05) based on least significance Difference (LSD). CV denotes coefficient of variation. BC = broadcasting.

### Panicle length

Panicle length is a key attribute of teff yield [31]. Statistical analysis revealed this trait was significantly influenced by variety, sowing method and seed rate, whereas their combined impact is non-significant (Table 1). The longest panicles (43.82 cm) were resulted from sowing teff at a rate of 10 kg ha^–1^, while the shortest panicles (41.61 cm) were from 20 kg ha^–1^ (Table 7). In general, a reduction of rate of sowing from 20 kg ha^–1^ to 10 kg ha^–1^ resulted in an increase of 2.21 cm in panicle length. This increased panicle length under lower seeding rate is attributed to the reduction in competition among plants, allowing more efficient use of growth resources. A significant increase in panicle length was recorded for teff as the sowing rate was lowered from 25 kg ha^–1^ to 10 kg ha^–1^ [32,33].

The present study revealed that the greatest panicle length was recorded in the variety *Quncho* (45.14 cm), followed by the varieties *Kora* (43.24 cm) and *Abolse* (39.63 cm) (Table 7). The variation in panicle length among varieties is due to their genetic differences, meaning that the differences in panicle length are attributed to heritable traits rather than to environmental factors [13]. also reported greater panicle length (48.34 cm) in the improved variety *Quncho* than in the local variety. Contrary [27], reported greater panicle length in the local variety (42.53 cm) than in the improved varieties *Kora* and *Quncho*. This discrepancy is likely driven by genotype-by-environment (G × E) interactions, where high phenotypic plasticity allows specific local landraces to exhibit pronounced organ elongation under distinct micro-climates or lower-input conditions.

As to the effect of the sowing method on panicle length in teff, the longest panicle (44.46 cm) was recorded in response to sowing teff at 20 cm row spacing. The traditional broadcasting method produced the shortest panicle length (41.13 cm) (Table 7). The variation between the longest and shortest panicle lengths could be attributed to the optimal nutrient utilization and partitioning of photosynthates under wider spacing. Increased competition for available resources restricts vegetative development, leading to a significant reduction in panicle length [24].

### Biomass yield

Biomass yield is important because farmers give a high value for teff not only to its grain but also to its straw. The main effects of variety, sowing method and seed rate significantly affected biomass yield (Table 1). Variety *Quncho* produced the maximum biomass yield (8.65 t ha^–1^), representing an increase of 18.98% over the local *Abolse* variety. Among the sowing method, row sowing of teff 15 cm apart between rows produced significantly a higher biomass yield (8.32 t ha^–1^). While the traditional broadcasting method produced the lowest amount of biomass yield (7.17 t ha^–1^) (Table 3). As the seed rate increased from 10 to 20 kg ha^–1^, biomass yield decreased significantly from 8.46 to 7.19 t ha^–1^ representing 15.01% reduction (Table 3).

The interaction effect of variety, sowing method, and the seed rate on biomass yield was also statistically significant (Table 1). Variety *Quncho* produced the highest biomass yield (10.76 t ha^–1^) when it was seeded at a rate of 10 kg ha^–1^ and 15 cm between rows, and the lowest biomass yield (6.00 t ha^–1^) was recorded from the local *Abolse* cultivar when it was broadcasted and sown at 20 kg ha^–1^ (Table 5). The results further revealed an increase in the aboveground biomass yield as the sowing rate decreased from 20 kg ha^-1^ to 10 kg ha^–1^ with 15 cm row spacing.

**Table 5:** Interaction effects of variety, sowing method and seed rate on teff aboveground biomass yield (BY) at Mojana Wodera Woreda.

	BY (t ha^–1^)			
Variety	Sowing method (cm)	Seed rate (kg ha^–1^)		
		**10**	**15**	**20**
*Quncho*	15	10.76^a^	9.33^bc^	7.8^efg^
	20	9.32^bc^	9.54^b^	8.0^def^
	BC	8.31^d^	7.65^ihgf^	7.20^j^
*Kora*	15	9.09^c^	8.05^def^	7.28^hij^
	20	8.33^d^	8.11^ed^	7.5^ghij^
	BC	7.30^hij^	7.20^j^	6.50^k^
*Abolse*	15	8.09^edf^	7.34^ghij^	7.19^j^
	20	7.7^efgh^	7.50^ghij^	7.23^j^
	BC	7.19^j^	7.19^j^	6.00^l^
LSD (5%)	0.43			
CV (%)	3.13			

Values in a column followed by the same letter are not significantly different at the 5% level of significance. where LSD = least significant difference and CV = coefficient of variation. BC = Broadcasting.

The relatively high biomass of the *Quncho* variety in the selected treatments in this study might be attributed to its biological yield potential, ease of agronomic management at a relatively low seed rate, and the effectual utilization of growth resources, which facilitated the growth of a plant. Consistent with this study [34], reported that the variety *Quncho* produced the highest aboveground biomass yield when sown at 15 cm row spacing and sowing rates of 10 kg ha^–1^. In general, despite the variety and sowing method, there was as significant decline in biomass yield as the seeding rate increased from 10 to 20 kg ha^–1^. This might be due to the direct relationship between biomass yield and factors such as plant height and tiller number, all of which are influenced by the seed rate, sowing method, and variety. The Quuncho variety produced the highest aboveground biomass yield when it was seeded at a row spacing of 15 cm [9,11].

### Lodging index (%)

The percentage of lodging in teff was affected by the main factors variety, sowing method, and seed rate (*P* < 0.01) (Table 1). Among the evaluated varieties, variety *Kora* showed superior lodging resistance, recording the lowest lodging percentage (30.33%), whereas variety *Quncho* was the most susceptible (38.18%). Regarding sowing method, sowing teff at a spacing of 15 cm significantly reduced lodging (27.18%) compared to broadcasting (38.87%). As the seed rate increased from 10 kg ha^–1^ to 20 kgha^–1^ lodging increased significantly from 27.18% to 38.87% (Table 3).

A significant three-way interaction effect (variety× sowing method ×seed rate) was observed for lodging percentage (P < 0.01) (Table 1). Broadcasting of *Quncho* cultivar at a sowing rate of 20 kg ha^–1^ produced the highest percentage in lodging index (44.00%). Sowing variety *Kora* at a row spacing of 15 cm combined with a seed rate of 10 kg ha^–1^ had the least value of lodging index (16.33%) (Table 6). The increased lodging percentage of the *Quncho* variety with the broadcast sowing method, particularly at seeding rates of 15 and 20 kg ha^-1^, could be attributed to greater competition for nutrients, moisture, and air, which was mainly due to the greater plant density at these sowing rates. This competition likely resulted in weaker, more succulent stems that are susceptible to damage from strong winds and rainfall. However, the stems of the teff varieties tested in this study, which were sown in rows at an ample seed rate, were strong enough to hold the weight of the grain heads [27]. Broadcasting of teff at higher seed rate increased the lodging percentage of teff [33].

**Table 6:** Interaction effects of variety, sowing method and seed rate on the logging index (%) of teff in the Mojana Wodera Woreda district 2021.

Variety	Sowing method (cm)	Seed rate (kg ha^–1^)		
		10	15	20
*Quncho*	15	29.33^i^	38.00^e^	40.00^c^
	20	31.67^g^	40.00^c^	42.33^b^
	BC	35.00^f^	43.33^ab^	44^a^
*Kora*	15	16.33^m^	31.00^gh^	32.00^g^
	20	20.00^l^	32.33^g^	36.00^f^
	BC	30.00^hi^	35.33^f^	40.00^c^
*Abolse*	15	24.33^k^	36.17^f^	35.00^f^
	20	27.00^j^	38.00^e^	38.33^de^
	BC	31.00^gh^	39.83 cd	42.17^b^
LSD (5%)	1.62			
CV (%)	2.85			

Values with the same letter are not significantly different at the 5% level of significance. Where LSD = least significant difference and CV = coefficient of variation. BC = broadcasting.

The broadcasting at higher seed rates resulted in more lodging compared to lower seed rate [32]. These findings suggest that the seed rate and row spacing are critical factors in determining the lodging percentage of teff crops, despite the variety of varieties used. Similarly, a significant lodging index was reported at a seeding rate of 25 kg ha^–1^ for Vertisols and Cambisols [10]. Additionally [35], reported that reducing the recommended teff seeding rate from 25 kg ha^–1^ to 10 kg ha^–1^ enhances the strength and lodging resistance of teff shoots. This contradicts the findings of [36], who reported a high lodging index (47%) at the lowest seeding rate of 10 kg ha^–1^.

### Grain yield

The impact of varying variety, sowing methods and seed rate and in teff crop affected grain yield significantly (Table 1). The maximum (2.47 t ha^–1^), medium (2.13 t ha^–1^), and minimum (1.95 t ha^–1^) yields were recorded for the *Quncho*, *Kora*, and *Abolse* varieties, respectively (Table 7). In general, the *Quncho* variety outperformed the local *Abolse* variety and the *Kora* variety by 21.05% and 8.45%, respectively. This difference might be attributed to differences in their genetic makeup. Similarly [35], reported that grain yield differences among different varieties of teff. Compared with local varieties, improved varieties of teff presented yield advantages of 16.57% and 13.61% from the *Kora* and *Quncho* varieties, respectively [27].

**Table 7:** Effects of variety, sowing method and seed rate on panicle length (cm) and grain yield of teff in Mojana Wodera Woreda.

Varieties	Panicle length(cm)	Grain yield (t ha^–1^)
*Quncho*	45.14 ^a^	2.47^a^
*Kora*	43.24 ^b^	2.13 ^b^
*Abolse* or Local	39.63 ^c^	1.95^c^
**Sowing method (cm)**		
15	42.42 ^b^	2.18^b^
20	43.24 ^b^	2.32^a^
BC	41.13 c	2.05^c^
**Seed rate (kg ha**^**-1**^)		
10	42.42 ^b^	2.22^a^
15	42.59^b^	2.27^a^
20	41.61^b^	2.05^b^
LSD (%)	0.16	0.09
CV (%)	3.72	8.11

Values with the same letter for the same parameter are not significantly different. LSD = least significant difference; CV = coefficient of variation. BC = broadcasting.

As to the impact of seed rate, 15 kg ha^–1^ produced the highest yield (2.27 t ha^–1^), while a seed rate of 20 kg ha^–1^ resulted in the lowest yield (2.05) (Table 7). This improvement at the lower seed rate in grain yield was attributed to the increased space available for teff tillers. Similar findings were also reported despite of the soil type [26]. Contradictory to this study sowing teff at highest seed rate (25 kg ha^–1^) produced the highest yield [9].

Sowing teff at 20 cm row spacing produced a significantly greater yield (2.32 t ha^-1^) which is 11.6% greater than the yield recorded from broadcasting, which produced the lowest yield (2.05 t ha^–1^) ^1^ (Table 7). The increase in grain yield because of wider spacing is resulted from a robust plant population by reducing intra-row competition. Similarly, a 27.06% greater teff yield was recorded with the row sowing method than with the broadcasting method [37].

## Conclusion

Teff is one of the most widely cultivated cereal crops in the lowlands of Ethiopia, including Mojana Wodera Woreda. Farmers in the study area produce teff both as food and cash crops under rain-fed conditions. The productivity of teff in the Amhara region and Mojana Wodera Woreda remains low, even though there is significant potential for increased production and yield. This is due to several constraints, such as the limited adoption of improved varieties and sowing methods (only 2% of farmers), lodging problems due to the inappropriate use of varieties and the seed rate. This study evaluated the interactive effects of seed rate, sowing method, and crop variety to identify optimal agronomic combinations for enhanced productivity of teff under local field conditions. The trial was conducted in 2022 at a training center in Mojana Wodera woreda, central Ethiopia using a Randomized Complete Block Design (RCBD) with three replications. The result revealed significant differences among the evaluated treatments, showing that agronomic performance of teff was strongly influenced by the management practices and variety choice. Specifically, variety *Quncho* produced the highest grain yield (2.47 t ha^–1^). In addition, sowing teff at a rate of 10 kg ha^–1^ and with a row spacing of 20 cm produced the highest grain yield of 2.22 t ha^–1^ and 2.32 t ha^–1^, respectively. In conclusion, the sowing method, seed rate and variety significantly affected teff growth and productivity. The row sowing of teff in this experiment enhanced field management and the plant population, reducing competition for growth resources by improving the yield and yield components of the crop. Low seed rates also resulted in relatively high grain yields. Among the varieties, *Quncho* and *Kora* consistently outperform the local *Abolse* variety, with *Quncho* showing the greatest yield advantage. Overall, optimizing these factors can lead to substantial improvements in the productivity. While this study provides a valuable insight to the agronomic practices of teff in Mojana Wodera woreda, a kely limitation is that the trial was conducted at a single location over a single cropping season. Because genotype by environment interaction (G × E) vary across years and location, future research should be conducted as multi-location and multi-year trials to test yield stability under fluctuating climatic conditions.

## Supporting information

S1 Data Raw data for variety, sowing rates, and methods on teff. This file contains the complete plot-level experimental data, including replications, variety, sowing methods, sowing rates, and corresponding yield measurements used in the statistical analysis.
(XLSX)

S2 Data Meteorological data containing monthly rainfall, maximum temperature, and minimum temperature during the experimental period. (XLSX)

S1 Code R script used for the statistical analysis of agronomic and yield data. (R)
