# Supplementary material for: Interactive effects of seed rate, sowing method, and variety on Teff (Eragrostis tef) yield, yield components, and lodging trait performance in the central highlands of Ethiopia
Source: PLoS One. 2026 Oct 12;21(10):e0360297. doi: 10.1371/journal.pone.0360297 (PMC13657313; doi:10.1371/journal.pone.0360297)

PLOS One

Genotypic Differences in Teff (Eragrostis tef) Yield Response to Sowing Rates and Methods in the Central Highlands of Ethiopia

--Manuscript Draft--

**Manuscript Number:**	PONE-D-26-15264
**Article Type:**	Research Article
**Full Title:**	Genotypic Differences in Teff (Eragrostis tef) Yield Response to Sowing Rates and Methods in the Central Highlands of Ethiopia
**Short Title:**	Genotype and methods of sowing on teff
**Corresponding Author:**	Fresew Belete, Ph.D<br>Debre Berhan University<br>Debre Berhan, ETHIOPIA
**Keywords:**	competition, lodging, panicle length, physiological maturity, seed
**Abstract:**	Teff (Eragrostis tef) occupies close to three million hectares of agricultural land in Ethiopia, nonetheless, realized yields remain substantially below the crop's inherent genetic yield potential. Thus, this study aimed to examine how different sowing rates and methods impacted productivity of teff varieties in Mojana Wodera Woreda. The treatments included three varieties of teff (namely, Quncho, Kora and Abolse (local variety), three sowing rates (10, 15 and 20 kg ha -1), and three sowing methods (15 cm and 20 cm spacing between rows and broad casting). Sowing variety quncho at a rate of 10 kg ha-1 with a row spacing of 15 cm produced the maximum amount of aboveground biomass yield (10.76 t ha-1). It also produced the largest panicle (45.14 cm) and yield (2.47 t ha-1). Furthermore, the maximum panicle length (44.46 cm) and yield (2.32 t ha-1) were recorded when teff was sown 20 cm apart between rows. The highest grain yield and largest panicle were when teff was seeded at 10 kg ha-1. Therefore, variety Quncho was the best for the study area, and sowing teff at a rate of 10 kg ha-1 with 20 cm spacing between rows enhanced its productivity.
**Order of Authors:**	Fresew Belete, Ph.D
	Yemanebirhane Tesfa
	Wondwosen Tena, Ph.D
**Additional Information:**	
**Question**	**Response**
**Financial Disclosure**<br><br>Enter a financial disclosure statement that describes the sources of funding for the work included in this submission. Review the submission guidelines for detailed requirements. View published research articles from *PLOS ONE* for specific examples.<br><br>This statement is required for submission and **will appear in the published article** if the submission is accepted. Please make sure it is accurate.	The author(s) received no specific funding for this work.

Powered by Editorial Manager® and ProduXion Manager® from Aries Systems Corporation

**Funded studies**<br>Enter a statement with the following details:<br>• Initials of the authors who received each award<br>• Grant numbers awarded to each author<br>• The full name of each funder<br>• URL of each funder website<br>• Did the sponsors or funders play any role in the study design, data collection and analysis, decision to publish, or preparation of the manuscript?<br><br>Did you receive funding for this work?	
**Competing Interests**<br><br>Use the instructions below to enter a competing interest statement for this submission. On behalf of all authors, disclose any competing interests that could be perceived to bias this work—acknowledging all financial support and any other relevant financial or non-financial competing interests.<br><br>This statement is required for submission and **will appear in the published article** if the submission is accepted. Please make sure it is accurate and that any funding sources listed in your Funding Information later in the submission form are also declared in your Financial Disclosure statement.<br><br>View published research articles from *PLOS ONE* for specific examples.	The authors have declared that no competing interests exist.

Powered by Editorial Manager® and ProduXion Manager® from Aries Systems Corporation

**NO authors have competing interests**<br><br>Enter: *The authors have declared that no competing interests exist.*<br><br>**Authors with competing interests**<br><br>Enter competing interest details beginning with this statement:<br><br>*I have read the journal's policy and the authors of this manuscript have the following competing interests: [insert competing interests here]*<br><br>* typeset	
**Ethics Statement**<br><br>Enter an ethics statement for this submission. This statement is required if the study involved:<br><br>• Human participants<br>• Human specimens or tissue<br>• Vertebrate animals or cephalopods<br>• Vertebrate embryos or tissues<br>• Field research<br><br>Write "N/A" if the submission does not require an ethics statement.<br><br>General guidance is provided below. Consult the submission guidelines for detailed instructions. **Make sure that all information entered here is included in the Methods section of the manuscript.**	N/A

**Format for specific study types**<br><br>**Human Subject Research (involving human participants and/or tissue)**<br>• Give the name of the institutional review board or ethics committee that approved the study<br>• Include the approval number and/or a statement indicating approval of this research<br>• Indicate the form of consent obtained (written/oral) or the reason that consent was not obtained (e.g. the data were analyzed anonymously)<br><br>**Animal Research (involving vertebrate animals, embryos or tissues)**<br>• Provide the name of the Institutional Animal Care and Use Committee (IACUC) or other relevant ethics board that reviewed the study protocol, and indicate whether they approved this research or granted a formal waiver of ethical approval<br>• Include an approval number if one was obtained<br>• If the study involved *non-human primates*, add *additional details* about animal welfare and steps taken to ameliorate suffering<br>• If anesthesia, euthanasia, or any kind of animal sacrifice is part of the study, include briefly which substances and/or methods were applied<br><br>**Field Research**<br><br>Include the following details if this study involves the collection of plant, animal, or other materials from a natural setting:<br>• Field permit number<br>• Name of the institution or relevant body that granted permission	
**Data Availability**<br><br>Authors are required to make all data underlying the findings described fully available, without restriction, and from the time of publication. PLOS allows rare exceptions to address legal and ethical concerns. See the PLOS Data Policy and FAQ for detailed information.	Yes - all data are fully available without restriction

Powered by Editorial Manager® and ProduXion Manager® from Aries Systems Corporation

A Data Availability Statement describing where the data can be found is required at submission. Your answers to this question constitute the Data Availability Statement and **will be published in the article**, if accepted.<br><br>**Important:** Stating 'data available on request from the author' is not sufficient. If your data are only available upon request, select 'No' for the first question and explain your exceptional situation in the text box.<br><br>Do the authors confirm that all data underlying the findings described in their manuscript are fully available without restriction?	
**Describe where the data may be found in full sentences. If you are copying our sample text, replace any instances of XXX with the appropriate details.**<br><br>• If the data are **held or will be held in a public repository**, include URLs, accession numbers or DOIs. If this information will only be available after acceptance, indicate this by ticking the box below. For example: *All XXX files are available from the XXX database (accession number(s) XXX, XXX.).*<br>• If the data are all contained **within the manuscript and/or Supporting Information files**, enter the following: *All relevant data are within the manuscript and its Supporting Information files.*<br>• If neither of these applies but you are able to provide **details of access elsewhere**, with or without limitations, please do so. For example:<br><br>*Data cannot be shared publicly because of [XXX]. Data are available from the XXX Institutional Data Access / Ethics Committee (contact via XXX) for researchers who meet the criteria for access to confidential data.*<br><br>*The data underlying the results presented in the study are available from (include the name of the third party*	All relevant data are within the manuscript and its Supporting Information files.

and contact information or URL).<br>• This text is appropriate if the data are owned by a third party and authors do not have permission to share the data.<br><br>* typeset	
Additional data availability information:	

Powered by Editorial Manager® and ProduXion Manager® from Aries Systems Corporation

Manuscript Click here to access/download;Manuscript;Manuscript.docx

Title: Genotypic Differences in Teff (*Eragrostis tef*) Yield Response to Sowing Rates and Methods in the Central Highlands of Ethiopia

Yemanebirhane Tesfa[1], Fresew Belete[1*], Wondwosen Tena[2]

[1]Department of Plant Sciences, College of Agriculture and Natural Sciences, Debre Berhan University

Email: Email: yemanebirehentesfa12@gmail.com

[1]Department of Plant Sciences, College of Agriculture and Natural Sciences, Debre Berhan University

Email: fresewbelete@dbu.edu.et

[2]Department of Plant Biology and Biodiversity Management, College of Natural and Computational Sciences, Addis Ababa University, Ethiopia.

Email: wondtena@gmail.com

* Corresponding author

Email: fresewbelete@dbu.edu.et

Abstract

Teff (*Eragrostis tef*) occupies close to three million hectares of agricultural land in Ethiopia, nonetheless, realized yields remain substantially below the crop's inherent genetic yield potential. Thus, this study aimed to examine how different sowing rates and methods impacted productivity of teff varieties in Mojana Wodera Woreda. The treatments included three varieties of teff (namely, Quncho, Kora and Abolse (local variety), three sowing rates (10, 15 and 20 kg ha^{-1}), and three sowing methods (15 cm and 20 cm spacing between rows and broad casting). Sowing variety quncho at a rate of 10 kg ha^{-1} with a row spacing of 15 cm produced the maximum amount of aboveground biomass yield (10.76 t ha^{-1}). It also produced the largest panicle (45.14 cm) and yield (2.47 t ha^{-1}). Furthermore, the maximum panicle length (44.46 cm) and yield (2.32 t ha^{-1}) were recorded when teff was sown 20 cm apart between rows. The highest grain yield and largest panicle were when teff was seeded at 10 kg ha^{-1}. Therefore, variety Quncho was the best for the study area, and sowing teff at a rate of 10 kg ha^{-1} with 20 cm spacing between rows enhanced its productivity.

Keywords: competition, lodging, panicle length, physiological maturity, seed

1. Introduction

Teff (*Eragrostis tef*) is a highly valued crop for its robust ecological tolerance and its foundational importance to the Ethiopia's cultural nutrition. It is an annual, self-pollinated, allotetraploid (20 chromosome pairs) C4 crop of the Poaceae family. The crop is domesticated in Ethiopia where its diversity is found [1]. Its value is the largest in the country in terms of both production and consumption [2,3]. The crop mainly used for Injera preparation, a soft, spongy flatbread that forms the cornerstone of Ethiopian cuisine. Teff is preferred by individual with wheat allergies as it is

gluten-free and is rich in different nutrients. Therefore, it can be considered an alternative to gluten-containing cereals such as wheat, barley and rye. Teff also reduce anemia because of its high iron content, as evidenced by the lack of cases of disease in areas where teff is commonly used [4]. Teffs grow in areas with altitudes between 1700 and 2800 m and is relatively affected by few pests and diseases [5]. Teff is a resilient crop to extreme weather, thriving under both drought and water-logged conditions. For these reasons, teff is considered a low-risk crop [2].

In Ethiopia, Teff ranks first in area coverage but has the lowest productivity. It accounts for 23.85% of the land allotted for cereals and 17.26% of the grain produced from cereals, and the national average yield remains low at 1.76 t ha^{-1} [6]. In Amhara region, teff ranks first in area coverage, with more than one million hectares, and is the most significant cereal cultivated by the majority of farmers [6]. With respect to the study area (Mojana Wodera), the North Showa Zone, teff covers more than 150 thousand hectares, and it accounts for 34.2% of the total allocated for cereal crops in the 2016 main cropping season [6]. Despite its importance, its productivity in the country, particularly in the Amhara region's Mojana Wodera Woreda, faces substantial production challenges that hinder its yield potential. The major challenges include lodging, the use of traditional sowing methods, improper seed rates, and the use of low yielding local varieties [7].

The seed size of teff is very small, and it creates a havoc during sowing, as it is difficult to control the distribution and population density. Farmers sow the crop at a higher seed rate than the recommended rate, mainly because of the use of unclean seeds with poor germination rates, which results in a 22% reduction in both straw and grain yield [8]. The seed rate and sowing method are essential agronomic aspects that demand careful consideration. The seed rate significantly

influences crop growth and development by determining plant density. Traditionally, teff is sown via the broadcasting method at 25-30 kg ha^{-1}. This resulted in a greater plant population per area, which increased nutrient, water, sunlight, and CO_2 competition. Additionally, compared with row spacing, broadcasting increases production costs because it requires a higher seed rate. Broadcasting also causes lodging, which is the main reason for the low yield of teff [7]. Lodging issues in teff can be minimized by using a lower seeding rate, planting in rows and selecting the proper variety. Row planting in teff has a greater yield advantage than does broadcasting [9]. Various studies have suggested different row spacings for teff. A spacing of 20 cm and 15 cm between rows was suggested by [10] and [11], respectively.

In the Amhara region, particularly in Mojana Wodera Woreda, farmers use broadcasting for sowing. However, there are no specific guidelines for row spacing or proper seeding rates for various teff varieties [7]. Moreover, farmers rely on local teff varieties, do not use row-planting techniques, and do not apply optimal seed rates to their farms. These practices often reduce crop yields. This research investigates how seeding rate and sowing method influence the productivity of different teff genotypes.

2. Materials and Methods

2.1. Study Area

The field experiment was done during 2022 in Mojana Wodera woreda at Zubaba Keble (70°55' N and 39°37' E) in central Ethiopia (fig 1). The woreda receives 852 mm of rainfall annually and is known for producing rain-fed teff in Ethiopia (Fig 2). The average temperatures of the woreda ranges between 12.5°C and 20.5°C. The soil type is mainly pure Vertisols, which occupy approximately 4045 ha.

Fig 1. Map of the study area

Fig 2. Rainfall, minimum and maximum temperatures during 2022 in Mojana wodera woreda

2.2. Experimental design and cultural practice

Three distinct teff genotypes: Abolse (a local landrace), kora and Quncho were evaluated against a three sowing rates (10, 15 and 20 kg ha^{-1}) and three methods of sowing. The later comprised a traditional broadcast method alongside tow row planting at a spacing of 0.15m and 0.2m, respectively. The study was a factorial design with three separate replications in a randomized complete block design (RCBD). A 3 m by 1.5 m plot was prepared. For row sowing treatments, each plot had 18 rows with 15 cm spacing and 13 rows with 20 cm spacing for each variety. The design employed 1 m interval between replications and 0.5 m between adjacent plots. Seeds were drilled in rows and broadcasted following the seed rate and sowing method treatments. NPSB fertilizer at a recommended rate (100 kg ha^{-1}) was applied at sowing, while 100 kg ha^{-1} urea was applied, with half of it applied 15–18 days after seeding and the rest applied at the crop tillering stage [12]. The plots were kept weed free. No insecticides or fungicides were used, as there were no insect or disease outbreaks.

2.3. Data Collection

The following measurements were taken including the days to 50% heading (D50H), days to 90% physiological maturity (D90M), plant height (PH), number of tillers per plant (NT), panicle length (PL), biomass yield(BY), and grain yield (GY). Lodging index (LI): the extent of lodging was assessed visually by observing the angle between the main stem's inclination and the vertical base. A scale of 1-5 was used: 1 (0-15°) indicates no lodging, 2 (15--30°) indicates 25% lodging, 3 (30-45°) indicates 50% lodging, 4 (45-60°) indicates 75% lodging, and 5 (60-90°) indicates 100% lodging [13].

2.4. Statistical analysis

A three way ANOVA was performed on the dataset to determine if there were variations among the teff genotypes in response to sowing rate and sowing method utilizing R statistical package Version R-4.05. Mean comparison was performed via the Duncan multiple range test at the 5% level of significance.

3. Results and Discussions

3.1. Days to 50% heading (DH)

The mean square analysis of variance confirmed that variety, row spacing, and seed rate independently impacted days to 50% heading significantly, their combined interactions (two-way and three-way) remained non-significant (Table 1). Significantly, longer days to heading (59.36 and 58.37 days) were recorded from the lower seeding rates (10 and 15 kg ha^{-1}), whereas the minimum number of days (57.66 days) was from the highest seeding rate (20 kg ha^{-1}) (Table 2). The earlier heading under higher seeding rates might be induced as a competitive stress response, causing the teff crop to enter the reproductive phase to ensure seed production before resources are exhausted. However, a lower seed rate reduces competition and enables plants to access

necessary growth resources, which leads to vegetative growth extension, resulting in a delay in the heading of the teff crop. An increase in planting density hastens the inflorescence emergence in rice plants [9]. In contrast to the findings of this study, [14] reported a significant ontogenetic delay in teff when it was sown at 20 kg ha $^{-1}$.

Table 1. Mean squares from the ANOVA for phenological, growth, and yield related traits of teff varieties ~~due to the~~ effects of the sowing rate and sowing technique in the Mojana Wodera

Source of variation	**Parameters**							
	D	PH	NT	PL	BY	GY	SY	LI
Rep (2)	26.43	5.07	0.53	11.34	0.31	0.21	0.26	1.51
V (2)	18.06*	3791**	11.26**	211.35**	13.48**	0.1.89**	20.38**	417.51**
SR (2)	19.69*	646.37**	5.52**	32.92**	11.04**	0.35*	1.39**	1071.73**
SM (2)	19.25*	562.94**	6.12**	76.35**	10.32**	0.49**	3.53**	288.86**
SR*SM (4)	1.59NS	122.07**	1.34*	1.06NS	0.89**	0.06NS	0.54*	11.72**
V*SR (4)	0.44NS	19.38*	0.58NS	0.83NS	0.57**	0.06NS	1.8**	10.85**
V*SM (4)	3.22NS	56.9***	0.02NS	0.71NS	0.39***	0.03NS	0.25NS	9.4***
V*SR*SM (8)	0.24NS	44.17**	0.15NS	0.55NS	0.4***	0.01NS	0.51**	5.61**
Error	2.11	5.84	0.34	2.52	0.07	0.04	0.11	0.98

Where Rep= replication; V= variety; SR= seed rate; and SM= sowing method, NS= non-significant

**= highly significant

With respect to variety, Kora took a significantly longer time (59.18 days) to heading than did the variety Quncho (58.63 days) and the local variety Abolse (57.57 days) (Table 2). The variation in heading date could be due to the genetic differences among the three cultivars in response to different seeding rates and planting methods. Similarly, earliness in days to heading of teff varies with cultivar.

With respect to the sowing method, the present study revealed that sowing teff with 20 cm row spacing significantly delays the number of days to heading compared with that with narrower 15 cm spacing and broadcasting (Table 2). The wider spacing and improved management within rows may have reduced intraspecific competition, allowing efficient utilization of growth resources. The earlier heading of teff plants observed in this study with narrow spacing may be due to the increased competition for resources in closer rows. This competition might have caused the plants to experience resource shortages, leading them to head earlier to escape stressful conditions rather than extending their vegetative growth and biomass accumulation. Additionally, genetic factors may play a role [7].

Table 2: Impact of different sowing methods and seed rates on days to heading of teff varieties in Mojana Wodera Woreda

Variety	Days to heading
Quncho	58.63^{b}
Kora	59.18^{a}
Abolse	57.57^{b}

Row spacing(cm)	
15	58.40[b]
20	59.33[a]
Broad casting	57.65[b]
Sowing rate (kg ha^{-1})	
10	59.36[a]
15	58.37[b]
20	57.66[b]
LSD (0.05)	0.79
CV (%)	2.49

Mean values sharing a common letter are non-significant (P>0.05) based on least significance Difference (LSD). CV denotes coefficient of variation.

3.2. Number of tillers

The mean square analysis demonstrated that tiller production per plant was significantly determined by variety, sowing technique and seed rate. Similarly, sowing methods and seed rate interactions also had notable effects on this trait (Table 1). The highest tillering capacity (7.09) was achieved through a seeding rate of 10 kg ha^{-1} combined with 20 cm row spacing. In contrast, broadcasting yielded to a significantly the lowest tiller count per plant (Table 3). This finding highlights a low seed rate and increased inter-row spacing optimize plant access to nutrients and light allowing plants to express their full tillering potential, while denser populations lead to nutrient stress that stunts tillering [15]. This result aligns with the findings of [16], who noted that

lowering teff seeding rate from 20 to10 kg ha^{-1} during broadcast conditions enhanced tiller production by alleviating spatial constraints. Similarly, [17] reported that increasing the seeding density reduced total and productive tillers in teff crops. Compared with the broadcasting method, the row sowing method results in significantly more tillers per plant [1]. In contrast, [18] confirmed that seeding rate had no significant effect on individual plant tillering, while broadcasting at different seed rates significantly influenced the total tiller populations compared to structured row arrangement.

Table 3: Tillering performance of teff as a function of row spacing and seed rate in Mojana Wodera Woreda district.

	Seed rate kg ha^{-1}		
Row spacing (cm)	10	15	20
15	6.2^{b}	5.56^{bcd}	5.22^{cd}
20	7.09^{a}	5.95^{bc}	5.67^{bcd}
BC	5.29^{cd}	5.61^{bcd}	4.98^{d}
CV%	13.76		
LSD (0.05)	0.741		

Mean values sharing a common letter are non-significant (P>0.05) based on least significance Difference (LSD). CV denotes coefficient of variation. BC= broadcasting.

3.3. Plant height

Plant height is a crucial growth attribute that influences the productivity of a crop, affecting both yield and straw. It is also positively correlated with increased production and productivity of crops [19]. The mean square analysis in this study revealed a difference in the height of teff due to sowing method, seeding rate, and variety, as well as three-way interactions (Table 1). Variety Quncho reached the tallest height (128.33 cm) when sown at 20 cm row spacing and a 10 kg ha^{-1} seed rate. However, the local Abolse cultivar produced the shortest value (84.83 cm) when the seed rate was doubled to 20 kg ha^{-1} ~~and rows were narrowed~~ to 15 cm (Table 4). The interaction effect of the Quncho variety, sown at 10 kg ha^{-1} with 20 cm between rows, resulted in a 43.5 cm increase in height compared with the local Abolse variety seeded at 20 kg ha^{-1} and 15 cm between rows (Table 4).

In general, plant height significantly increased as the seeding rates were lowered from 20 kg ha^{-1} to 10 kg ha^{-1} during broadcast sowing, as the additional space between plants allowed for better growth. These findings suggest that using lower seed rates with the drilling method resulted in taller teff plants. This is corroborated by [14], who reported 8.1 cm height advantage when using 5 kg ha^{-1} rate versus 25 kg ha^{-1} seeding rate. Wider row spacing at constant seed rates tended to produce taller plants [16].

The variation in teff stature among different cultivars might also be attributed to their distinct genomic compositions. A similar result was reported by [7], ~~where~~ compared ~~with the~~ other tested varieties, Quncho had the tallest plant height (130.94 cm). However, [20] reported a different result where the local variety surpassed the height of Quncho, which exhibited a comparatively shorter stature in that specific environment.

Table 4: Interaction effects of variety, sowing method, and seed rate on plant height (cm) of teff in Mojana Wodera Woreda

		Seed rate (kg ha^{-1})		
Variety	Row spacing (cm)	10	15	20
Quncho	15	122[b]	116[dc]	110.8[e]
	20	128.33[a]	117.67[c]	114.2[dce]
	Broadcast	112[e]	112[e]	98.77[hi]
Kora	15	111.2[e]	94.17[j]	99.5[ghi]
	20	111.1[e]	112.4[de]	106.13[f]
	Broadcast	100.97[gh]	105.867[f]	96.17[ji]
Abolse	15	95.93ij	88[k]	84.83[k]
	20	103.33[gf]	93.47[j]	86.9[k]
	Broadcast	86.9[k]	93[j]	86.57[k]
LSD (5%)		3.95		
CV (%)		5.34		

Mean values sharing a common letter are non-significant (P>0.05) based on least significance Difference (LSD). CV denotes coefficient of variation.

3.4. Panicle length

Panicle length is a key attribute of teff yield [21]. Statistical analysis revealed this trait was significantly influenced by variety, sowing rate, and sowing method, whereas their combined

impact is non-significant (Table 1). The longest panicles (43.82 cm) were resulted from sowing teff at a rate of 10 kg ha^{-1}, while the shortest panicles (41.61 cm) were from 20 kg ha^{-1} (Table 7). In general, a reduction of rate of sowing from 20 kg ha^{-1} to 10 kg ha^{-1} resulted in an increase of 2.21 cm in panicle length. This increased panicle length under lower seeding rate is attributed to the reduction in competition among plants, allowing more efficient use of growth resources. A significant increase in panicle length was recorded for teff as the sowing rate was lowered from 25 kg ha^{-1} to 10 kg ha^{-1} [22,23].

The present study revealed that the greatest panicle length was recorded in the variety Quncho (45.14 cm), followed by the varieties Kora (43.24 cm) and Abolse (39.63 cm) (Table 7). The variation in panicle length among varieties is due to their genetic differences, meaning that the differences in panicle length are attributed to heritable traits rather than to environmental factors. [7] also reported greater panicle length (48.34 cm) in the improved variety Quncho than in the local variety. Contrary, [20] reported greater panicle length in the local variety (42.53 cm) than in the improved varieties Kora and Quncho.

As to the effect of the sowing method on panicle length in teff, the longest panicle (44.46 cm) was recorded in response to sowing teff at 20 cm row spacing. The traditional broadcasting method produced the shortest panicle length (41.13 cm) (Table 7). The variation between the ~~highest~~ and ~~lowest~~ panicle lengths could be attributed to the optimal nutrients and ~~greater row spacing~~, ~~which resulted in increased intraspecific competition among the plants~~. A decrease in panicle length is caused by the increasing competition at intermediate resources [19].

3.5. Biomass yield

Biomass yield is important because farmers give a high value for teff not only to its grain but also to its straw. The mean square analysis revealed that the teff biomass yield was highly significantly impacted by the combined effect of the seeding rate, sowing method, and variety (Table 1). Variety Quncho produced the highest biomass yield (10.76 t ha^{-1}) when it was seeded at a rate of 10 kg ha^{-1} and 15 cm between rows, and the lowest biomass yield (6.00 t ha^{-1}) was found for the local Abolse cultivar when it was broadcasted and sown at 20 kg ha^{-1} (Table 5). The results further revealed an increase in the aboveground biomass yield as the sowing rate decreased from 20 kg ha^{-1} to 10 kg ha^{-1} with 15 cm row spacing.

The relatively high biomass of the Quncho variety in the selected treatments in this study might be attributed to its biological yield potential, of the ease of agronomic management at a relatively low seed rate, and the effectual utilization of growth resources, which facilitate the growth of a plant. Consistent with this study,[24] reported that the variety Quncho produced the highest aboveground biomass yield when sown at 15 cm row spacing and sowing rates of 10 kg ha^{-1}. In general, despite the variety and sowing method, there was as significant decline in biomass yield as the seeding rate increased from 10 to 20 kg ha^{-1}. This might be due to the direct relationship between biomass yield and factors such as plant height and tiller number, all of which are influenced by the seed rate, sowing method, and variety. The Quuncho variety produced the highest aboveground biomass yield when it was seeded at a row spacing of 15 cm [1,4].

Table 5: Interaction effects of variety, seed rate and sowing method on teff above gournd biomass yield (AGBY) and straw yield (SY) at Mojana Wodera Woreda

		AGBY(t ha^{-1})		
Variety	row spacing	seed rate(kg ha^{-1})		
		10	15	20
Quncho	15	10.76^{a}	9.33^{cb}	7.8^{egf}
	20	9.32^{cb}	9.54^{b}	8.0^{edf}
	Broadcast	8.31^{d}	7.65^{ihgf}	7.20^{j}
Kora	15	9.09^{c}	8.05^{edf}	7.28^{ihj}
	20	8.33^{d}	8.11^{ed}	7.5^{ihgj}
	Broadcast	7.30^{ihj}	7.20^{j}	6.50^{k}
Abolse	15	8.09^{edf}	7.34^{ihgj}	7.19^{j}
	20	7.7^{ehgf}	7.50^{ihgj}	7.23^{j}
	Broadcast	7.19^{j}	7.19^{j}	6.00^{l}
LSD (5%)			0.43	
CV (%)			3.36	

Values in a column followed by the same letter are not significantly different at the 5% level of significance. where LSD = least significant difference and CV = coefficient of variation.

3.6. Lodging index (%)

The percentage of lodging in teff was affected by the individual factors as well as by the combination of the factors variety, seed rate and sowing method significantly (Table 1). Broadcasting of Quncho cultivar at a sowing rate of 20 kg ha^{-1} produced the highest percentage in lodging index (44.00%). Sowing variety Kora at a row spacing of 15 cm combined with a seed rate of 10 kg ha^{-1} had the least value of lodging index (16.33%) (Table 6). The increased lodging percentage of the Quncho variety with the broadcast sowing method, particularly at seeding rates of 15 and 20 kg ha^{-1}, could be attributed to greater competition for nutrients, moisture, and air, which was mainly due to the greater plant density at these sowing rates. This competition likely resulted in weaker, more succulent stems that are susceptible to damage from strong winds and rainfall. However, the stems of the teff varieties tested in this study, which were sown in rows at ~~an ample~~ seed rate, were strong enough to hold the weight of the grain heads [20]. Broadcasting of teff at higher seed rate increased the lodging percentage of teff [23].

The broadcasting of ~~teff resulted in higher lodging rates when higher seed rates were used than when lower seed rates were used~~ [22]. The present study further revealed that varietal differences did not influence the lodging percentage, as all the varieties were susceptible to various treatments. These findings suggest that the seed rate and row spacing are critical factors in determining the lodging percentage of teff crops, despite the variety of varieties used. Similarly, a significant lodging index was reported at a seeding rate of 25 kg ha-1 for Vertisols and Cambisols [3]. Additionally, [25] reported that reducing the recommended teff seeding rate from 25 kg ha^{-1} to 10 kg ha^{-1} enhances the strength and lodging ~~ability~~ of teff shoots. This contradicts the findings of [26], who reported a high lodging index (47%) at the lowest seeding rate of 10 kg ha^{-1}.

Table 6: Interaction effects of variety, seed rate and sowing method on the logging index (%) of teff in the Mojana Wodera Woreda district 2021.

Variety	Row spacing (cm)	Seed rate (kg ha^{-1})		
		10	15	20
Quncho	15	29.33^{i}	38.00^{e}	40.00^{c}
	20	31.67^{g}	40.00^{c}	42.33^{b}
	Broad cast	35.00^{f}	43.33^{ab}	44^{a}
Kora	15	16.33^{m}	31.00^{hg}	32.00^{g}
	20	20.00^{l}	32.33^{g}	36.00^{f}
	Broad cast	30.00^{hi}	35.33^{f}	40.00^{c}
Abolse	15	24.33^{k}	36.17^{f}	35.00^{f}
	20	27.00^{j}	38.00^{e}	38.33^{de}
	Broad cast	31.00^{hg}	39.83^{cd}	42.17^{b}
LSD (5%)			1.62	
CV (%)			7.1	

Values with the same letter are not significantly different at the 5% level of significance. Where LSD = least significant difference and CV = coefficient of variation.

3.7. Grain yield

The impact of varying sowing methods, seed rates, and varieties in teff crop affected grain yield were significant (Table 1). The maximum teff (2.47 t ha^{-1}), medium (2.13 t ha^{-1}), and minimum (1.95 t ha^{-1}) teff yields were recorded for the Quncho, Kora, and Abolse varieties, respectively

(Table 7). In general, the Quncho variety outperforms the local Abolse variety and the Kora variety by 21.05% and 8.45%, respectively. This difference might be attributed to differences in their genetic makeup. Similarly, [25] reported that grain yield differences among different varieties of teff. Compared with local varieties, improved varieties of teff presented yield advantages of 16.57% and 13.61% from the Kora and Quncho varieties, respectively [20].

As to the impact of seed rate, 15 kg ha^{-1} produced the highest yield (2.27 t ha^{-1}), while a seed rate of 20 kg ha^{-1} resulted in the lowest yield (2.05) (Table 7). This improvement at the lower seed rate in grain yield was attributed to the increased space available for teff tillers. Similar findings were also reported despite of the soil type [16]. Contradictory to this study sowing teff at highest seed rate (25 kg ha^{-1}) produced the highest yield [1].

Sowing teff at 20 cm row spacing produced a significantly greater yield (2.32 t ha^{-1}) which is 11.6% greater than the yield recorded from broadcasting, which produced the lowest yield (2.05 t ha^{-1}) [1] (Table 7). The increase in grain yield because of wider spacing is resulted from a robust plant population by reducing intra-row competition. Similarly, a 27.06% greater teff yield was recorded with the row sowing method than with the broadcasting method [27].

Table 7: Effects of variety, seed rate and sowing method on panicle length (cm) and grain yield of teff in Mojana Wodera Woreda.

Varieties	Panicle length(cm)	Grain yield (t ha^{-1})
Quncho	45.14 ^{a}	2.47^{a}
Kora	43.24 ^{b}	2.13 ^{b}

Abolse or Local	39.63 [c]	1.95[c]
Row space (cm)		
15	42.42 [b]	2.18[b]
20	43.24 [b]	2.32[a]
BC	41.13 c	2.05[c]
Seed rate (kg ha^{-1})		
10	42.42 [b]	2.22[a]
15	42.59[b]	2.27[a]
20	41.61[b]	2.05[b]
LSD(%)	0.16	3.72
CV(%)	3.72	8.9

Values with the same letter for the same parameter are not significantly different. LSD = least significant difference; CV= coefficient of variation.

4. Conclusion

Teff is one of the most widely cultivated cereal crops in the lowlands of Ethiopia, including Mojana Wodera Woreda. Farmers in the study area produce teff both as food and cash crops under rain-fed conditions. The productivity of teff in the Amhara region and Mojana Wodera Woreda remains low, even though there is significant potential for increased production and yield. This is due to several constraints, such as the limited adoption of improved varieties and sowing methods (only 2% of farmers), ~~logging~~ problems due to the ~~appropriate~~ use of varieties and the seed rate. In conclusion, the sowing method, seed rate and variety significantly affected teff growth and productivity. The row sowing of teff in this experiment enhanced field management and the plant population, reducing competition for growth resources by improving the yield and yield

components of the crop. Low seed rates also resulted in relatively high grain yields. Among the varieties, Quncho and Kora consistently outperform the local Abolse variety, with Quncho showing the greatest yield advantage. Overall, optimizing these factors can lead to substantial improvements in the productivity.

References

1. Reda A, Dechassa N, Assefa K. Evaluation of seed rates and sowing methods on growth, yield and yield attributes of tef [Eragrostis tef (Zucc.) Trotter] in Ada District, East Shewa, Ethiopia. Agric Environ Sci. 2018;18(1):34–49.

2. Minten B, Tamru S, Engida E, Kuma T. Ethiopia's value chains on the move: The case of teff. Ethiop Strateg Support Progr II Work Pap. 2013;52:1–26.

3. Tesfay T, Gebresamuel G. Agronomic and economic evaluations of compound fertilizer applications under different planting methods and seed rates of tef [Eragrostis tef (Zucc.) Trotter] in Northern Ethiopia. J Drlylands. 2016;6(1):409–22.

4. Assefa K, Aliye S, Belay G, Metaferia G, Tefera H, Sorrells ME. Quncho: the first popular tef variety in Ethiopia. In: Sustainable Intensification. Routledge; 2012. p. 25–34.

5. Beyene G, Chauhan RD, Villmer J, Husic N, Wang N, Gebre E, et al. CRISPR/Cas9-mediated tetra-allelic mutation of the 'Green Revolution'SEMIDWARF-1 (SD-1) gene confers lodging resistance in tef (Eragrostis tef). Plant Biotechnol J. 2022;20(9):1716–29.

6. CSA. Central Statistical Agency (CSA). Agricultural sample survey for the 2017/2018 crop season. Volume I Report on Area and Production of Major Crops Private Peasant Holdings,

Meher Season. Statistical bulletin, FDRE/CSA, Addis Ababa, Ethiopia. 2019;

7. Negash W, Menzir A, Kassaye M. Effect of row spacing on yield and yield components of teff [Eragrostis tef (Zucc.) Trotter] varieties in Gonji Kolela district, North Western Ethiopia. J Biol Agric Healthc. 2017;7(23):35–43.

8. Tefera H, Belay G, Sorrells M. Narrowing the Rift. Tef research and development. 2001;

9. Kitata S, Abera T. Effects of Seed Rate and Row Spacing on Phenology, Growth, Yield and Economic Feasibility of Teff (Eragrostis tef [Zucc.] Trotter.) Production in Kiltu Kara District of Western Ethiopia Kiltu Kara District Agriculture and Natural Resource Office. Am J Sustain Agric. 2020 Oct;20:165–75.

10. Alemat E, Kidu G, Mihreteab H, Haftamu H. Determination of the optimum population density of seedlings during transplanting for the productivity improvement of Tef [Eragrostis tef (Zucc.)] Trotter] in the Central zone of Tigray, Ethiopia. J Biol Agric Healthc. 2016;6(1).

11. Asargew F, Bitew Y, Asfaw M, Liben M, Getahun W. Row spacing and fertilizer rate on yield and yield components of tef (Eragrostis teff (Zucc.) Trotter) under transplanting planting method. J Biol Agric Healthc. 2014;4(15):133–7.

12. Hameso H, Tefera T, Tadewos T. Effects of Lime and NPSB Fertilizer Rates on Yield Attributes and Yield of Wheat, and Soil Properties in Harbagona District of Sidama Region, Ethiopia. Ethiop J Agric …. 2023;33(3):58–77.

13. Girma D. The Relationships between Stem Characters and Lodging Tolerance in Tef

(Eragrostis tef) Genotypes. Ethiop J Agric Sci. 2019;29(2):59–70.

14. Abebe B, Abebe A. Effect of seed rate on yield and yield components of tef [(Eragrostic tef) Trotter] at Shebedino, Southern Ethiopia. J Nat Sci Res www iiste org. 2016;6(21).

15. Farooq M, Shahzad MA, Basra AS, Basharat AS. Direct seeding method popular among rice farmers, DAWN Group of News letter. Sri Lanka. 2006;89(2):11–28.

16. Dereje G, Adisu T, Dimberu A. Influence of seed rate and row spacing on growth and yield of tef (Eragrostis tef) production at assosa, Benishangul-Gumuz regional state, Western Ethiopia. Adv Crop Sci Technol. 2018;6:1–3.

17. Soomro UA, Rahman MU, Odhano EA, Gul S, Tareen AQ. Effects of sowing method and seed rate on growth and yield of wheat (Triticum aestivum). World J Agric Sci. 2009;5(2):159–62.

18. Adam MY. Effect Of Seed Rate And Nitrogen On Growth And Yield Of Teff Grass (Eragrostis Teff (Zucc.) Trotter. University of Khartoum; 2004.

19. Biya M. Response of Teff (Eragrostistef (Zucc) Trotter) to Seeding Rate and Row Spacing at Tiro-Afeta District, Southwestern Ethiopia. Int J Res Stud Sci Eng Technol. 2020;7(8):22–30.

20. Abera T, Gemechu D. Effect of Varieties and Seed Rate on Yield and Yield Components of Teff (Eragrostis tef) in Ambo District, Oromia Region, Western Ethiopia. World J Agric Sci. 2020;16(3):170–9.

21. Ketema HTS, Tesemma T. Variability, heritability and genetic advance in tef (Eragrostis

tef (Zucc.) Trotter) cultivars. Trop Agric. 1990;

22. Shiferaw T. Effects of inorganic fertilizer types and sowing methods of variable seed rates on yield and yield components of Tef [Eragrostis tef (Zucc.) Trotter]. Ada'a Woreda Cent Ethiop M Sc Theesis Haramaya Univ. 2012;1–69.

23. Sahle S, Altaye T. Effects of sowing methods and seed rates on yield components and yield of tef in soro woreda, hadya zone, southern Ethiopia. J Nat Sci Res. 2016;6(19):109–14.

24. Biya M. PP 22-30 ISSN 2349-476X International Journal of Research Studies in Science, Engineering and Technology V7 • I8 •. Vol. 7, International Journal of Research Studies in Science, Engineering and Technology. 2020.

25. Yihun YM, Schultz B, Haile AM, Erkossa T. Optimizing Teff productivity in a water stressed region of Ethiopia. In: ICID 21st International Congress on Irrigation and Drainage, Thehran, Iran, 15-20 October, 2011. 2011. p. 285–96.

26. Abebe B, Workayehu T. Effect of method of sowing and time of di-ammonium phosphate (dap) fertilizer application, on yield and yield components of tef (Eragrostis tef) Trotter) at Shebedino, Southern Ethiopia. Adv Crop Sci Technol. 2015;3:168.

27. Mengie Y, Assefa A, Jenber AJ. Sowing methods and seeding rates effects on yield and yield components of Tef (Eragrostis tef [Zucc.] Trotter) at Adet, North West Ethiopia. Heliyon. 2021;7(3).

Click here to access/download;Figure;Figures_page1_image2.tif

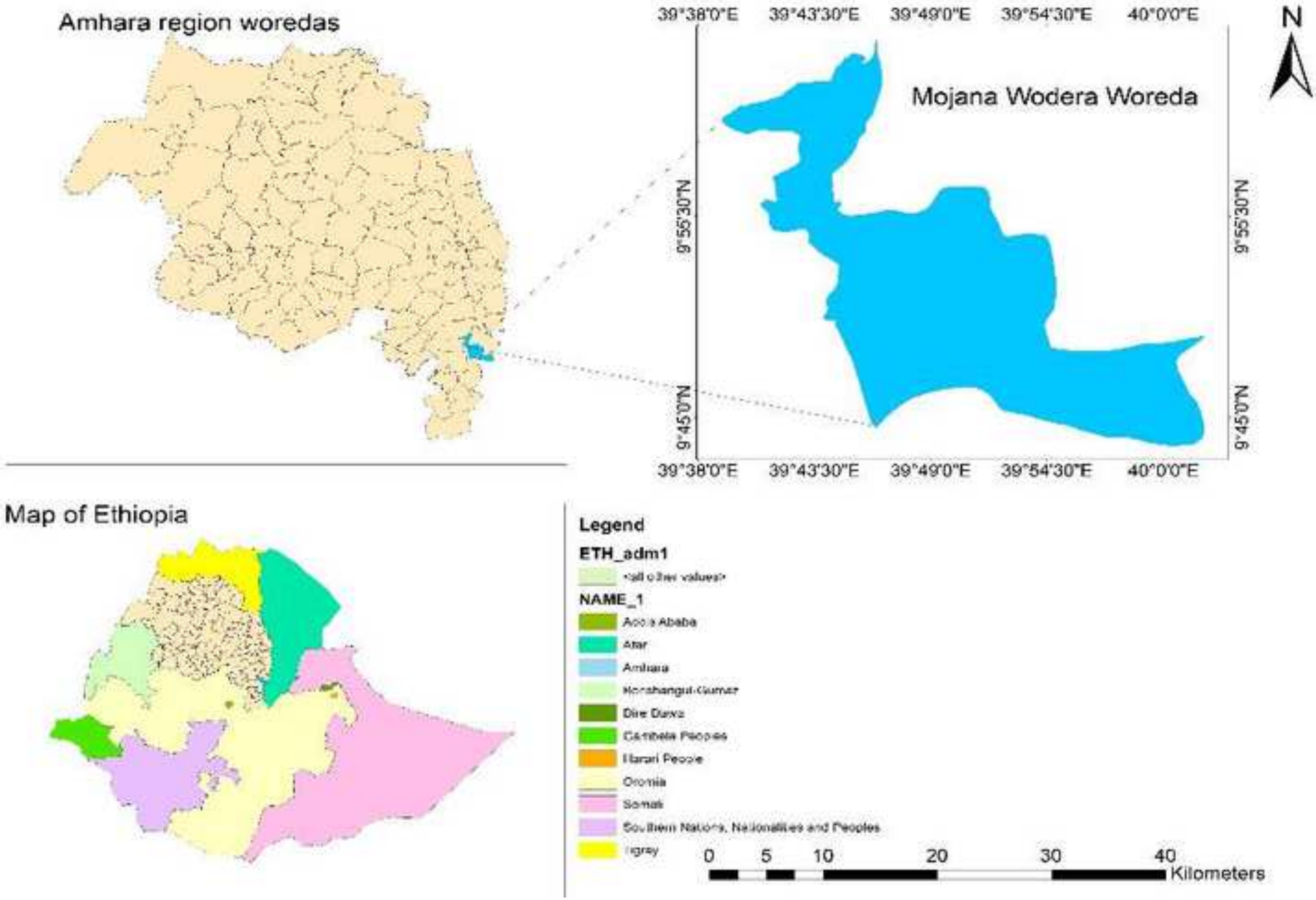

Figure 2

Click here to access/download;Figure;Fig 2.TIF

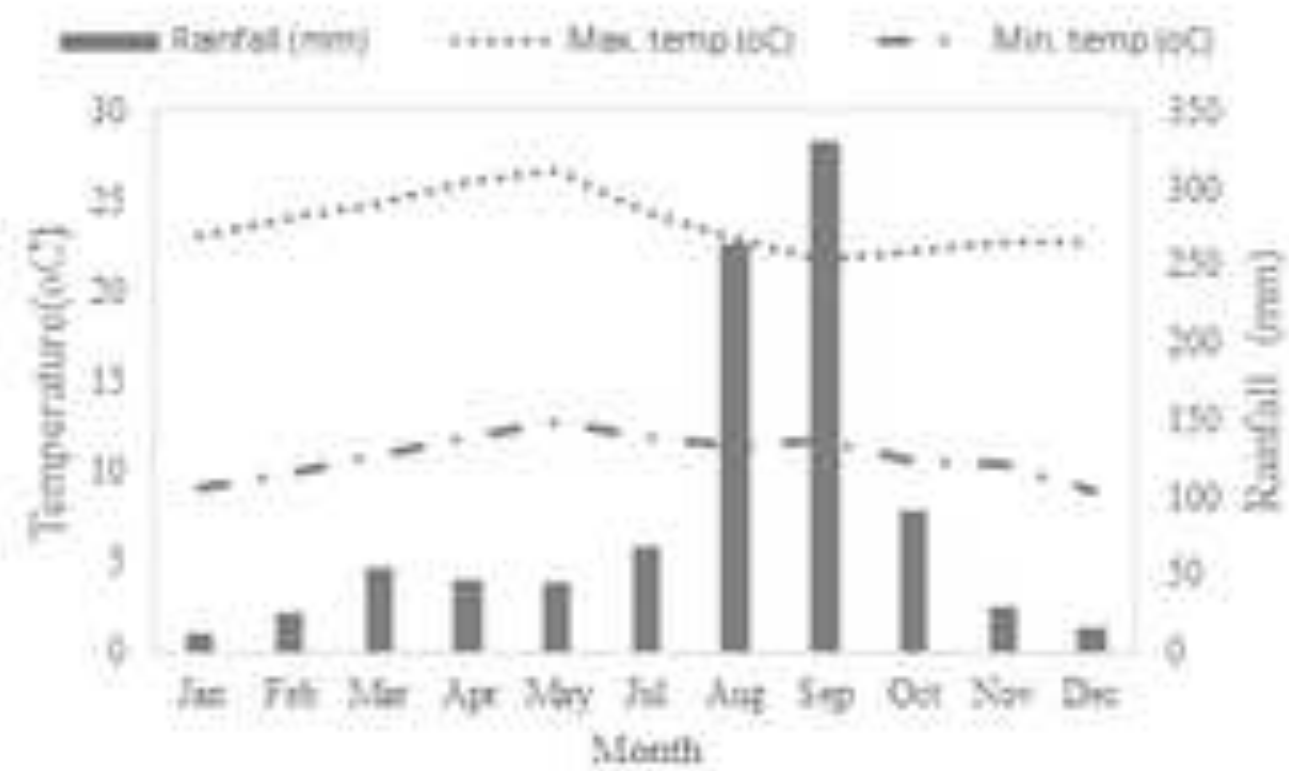

Supplement: Supplementary file 1 [file pone.0360297.s005.pdf]
