# Supplementary material for: Interactive effects of seed rate, sowing method, and variety on Teff (Eragrostis tef) yield, yield components, and lodging trait performance in the central highlands of Ethiopia
Source: PLoS One. 2026 Oct 12;21(10):e0360297. doi: 10.1371/journal.pone.0360297 (PMC13657313; doi:10.1371/journal.pone.0360297)
Supplement: Supplementary file 2 [file pone.0360297.s004.docx]

**General comments**

Generally, the manuscript is written well. However, revision is important to improve the clarity and novelty of the study

**Major comments**

**Abstract**

Clearly explain the results

**Conclusion**

In conclusion, clearly explain the presented result and what it means. Readers expect to see the objective of the study and the key methodology that was not explained.

**Minor comments**

To the introduction, add the world and continental production status

Explain the source of those two improved varieties under the methodology

Clearly explain the previous finding by including where it was conducted and its gap to increase reliability of this study

This study was only one location and one season, what would be your suggestion for future studies

**Specific comments**

Page 2, line 22: describe whether it is panicle length or panicle number

Page 2, Line 28: You concluded 10kg per ha rate by 20cm spacing as better productivity for Quncho variety. However, the result you wrote on Line 25 for the same variety Quncho showed better yield and panicle length at 10kg per ha and 15 cm spacing (e.g. 2.47 yield is higher than 2.32 per ha)

Page 2, Line 34: Cite statement with the chromosome number

Page 3, Line 50 to 52: use data of recent cropping season, 2016 is old.

Page 5, Line 90 to 91: When you look at the statement started from the later comprised…, some thing is missing.
