# Supplementary material for: Interactive effects of seed rate, sowing method, and variety on Teff (Eragrostis tef) yield, yield components, and lodging trait performance in the central highlands of Ethiopia
Source: PLoS One. 2026 Oct 12;21(10):e0360297. doi: 10.1371/journal.pone.0360297 (PMC13657313; doi:10.1371/journal.pone.0360297)
Supplement: Supplementary file 3 [file pone.0360297.s006.docx]

**Subject: Response to Reviewers for PONE-D-26-15264 Manuscript**

Title: “Genotypic Differences in Teff (*Eragrostis tef*) Yield Response to Sowing Rates and Methods in the Central Highlands of Ethiopia”

Dear Academic Editor,

Thank you very much for giving us the opportunity to revise our manuscript. We also wish to express our sincere gratitude to the reviewers for their constructive feedback and insightful suggestions, which have helped us significantly improve the quality of our manuscript.

Please note that all page and line numbers referenced in our responses below correspond to the marked-up version of the manuscript (**Revised Manuscript with Track Changes)**.

Below, we provide a point-by-point response to each comment raised by the Academic Editor and the reviewers.

**Section 1. Response to Academic Editor**

**Comment 1:**  *Please ensure that your manuscript meets PLOS ONE's style requirements, including those for file naming*

**Response**: We have thoroughly reviewed the journal’s formatting guidelines and updated the manuscript accordingly. All file names, heading structures, table and citation formats have been adjusted to comply with the journal's requirements. **Location: Throughout the manuscript**

**Comment 2:** *In your Methods section, please provide additional information regarding the permits you obtained for the work.*

**Response:** We have updated the Methods section to state explicitly that no specific permits were required for the site used for this study. **Location: Materials and Methods, Page 6-7, Lines 139-142**

**Comment 3:** *Please note that PLOS One has specific guidelines on code sharing for submissions in which author-generated code underpins the findings in the manuscript. In these cases, we expect all author-generated code to be made available without restrictions upon publication of the work.*

**Response:** We have complied fully with PLOS ONE’s Data Availability and Code Sharing policies. All underlying raw data and author-generated R code supporting the manuscript’s findings have been made publicly available without restriction to facilitate reproducibility and reuse.

1. Raw Data: The minimal dataset containing all recorded observations for the evaluated traits (*plant height, days to heading, number of tillers, panicle length, lodging index, biomass yield, and grain yield*) has been provided as Supporting Information **(S1 Data).**
2. Raw Data: the minimal dataset containing rainfall, maximum and minimum temperature has been provided as Supporting Information **(S2 Data)**
3. Analysis Code: The full R script used for processing the data, fitting the three-factor ANOVA models, and executing Fisher’s LSD post-hoc mean separations has been provided as Supporting Information **(S1 Code).**

**Comment 4:** *Please upload a new copy of Figure 1 as the detail is not clear*

**Response:** We apologize for the low resolution of Figure 1 in the previous submission. We have regenerated Figure 1 at high resolution (300 DPI) to ensure all details and text are clear and legible, in accordance with PLOS ONE's figure specifications.

**Comment 5:** *Please confirm at this time whether or not your submission contains all raw data required to replicate the results of your study...*

**Response:** We confirm that our submission contains the complete "minimal data set" required to fully replicate all statistical analyses, means, figures, and study findings reported in the manuscript, without restrictions.

All raw, individual observations behind the reported means and statistical outputs for all evaluated factors (variety, sowing method, and seed rate) and dependent traits (plant height, days to heading, number of tillers, panicle length, lodging index, biomass yield, and grain yield) have been compiled and uploaded as Supporting Information **(S1 Data)**. The graph data points for rainfall, maximum and minimum temperature was uploaded as **(S2 Data)**. Additionally, the R script used to analyze these data is uploaded as **(S1 Code).**

**Comment 6:** *If the reviewer comments include a recommendation to cite specific previously published works...*

**Response:** We note the journal's policy regarding citation recommendations. None of the reviewers requested or recommended the citation of specific previously published works for this manuscript.

**SECTION 2: RESPONSE TO REVIEWER #2**

**Comment 1: “***Abstract: Clearly explain the results”*

**Response:** We have revised the Abstract to clearly present numerical findings, treatment comparisons, and exact percentage/yield advantages rather than generic descriptions. **Location: Abstract, Page 2, Lines 32-45.**

**Comment 2:** *“Conclusion: In conclusion, clearly explain the presented result and what it means. Readers expect to see the objective of the study and the key methodology that was not explained.”*

**Response:** We have revised the Conclusion section to explicitly state the objective of the study, summarize the key methodology and synthesize the main agronomic findings. **Location: Conclusion, Page 22-23, Lines 441-449**.

**Minor Comment 1: “***To the introduction, add the world and continental production status”*

**Response:** We have added recent global and continental teff production status to the Introduction to properly contextualize the crop's global and regional economic importance. **Location: Introduction, Page 3, Lines 54-62.**

**Minor Comment 2:** *Explain the source of those two improved varieties under the methodology*

**Response:** We added the source for the improved teff varieties under the Materials and Methods section. **Location: Materials and Methods, Page 7, Lines 151-154.**

**Minor Comment 3:** *Clearly explain the previous finding by including where it was conducted and its gap to increase reliability of this study*

**Response:** We have revised the Introduction section to explicitly detail previous findings, clearly stating the geographic locations and agro-ecological of the studies were conducted. Furthermore, we have highlighted the specific research gaps; regarding variety, seed rate and sowing method interactions. **Location: Introduction, Pages 5–6, Lines 109-126.**

**Minor Comment 4:** *This study was only one location and one season, what would be your suggestion for future studies*

**Response:** We explicitly acknowledged this single-location, single-season study limitation in the Conclusion sections, adding a recommendation for multi-location and multi-year trials across contrasting agro-ecological zones. **Location: Conclusion, Pages 23, Lines 456-460.**

**Specific Comment 1:** *Page 2, line 22: describe whether it is panicle length or panicle number*

**Response:** We confirm that the measured parameter is **panicle length (cm)**

**Specific Comment 2 :** *Page 2, Line 28: You concluded 10kg per ha rate by 20cm spacing as better productivity for Quncho variety. However, the result you wrote on Line 25 for the same variety Quncho showed better yield and panicle length at 10kg per ha and 15 cm spacing (e.g. 2.47 yield is higher than 2.32 per ha)"*

**Response**: We have clarified the reporting of our statistical results to address this area of potential confusion. The value of 2.32 t ha⁻¹ represents the main effect for the sowing method, whereas 2.47 t ha⁻¹ represents the yield for the *Quncho* variety. We have updated the text on Page 2 to explicitly distinguish treatment means. **Location: Abstract, Pages 2, Lines 43–46.**

**Specific Comment 3:** *Page 2, Line 34: Cite statement with the chromosome number*

**Response:** We added the official citation supporting the chromosome number of teff (*Eragrostis tef,*) at this statement. **Location: Introduction, Page 3, Line 66.**

**Specific Comment 4:** *Page 3, Line 50 to 52: use data of recent cropping season, 2016 is old*

**Response:** We updated the statistical data to reflect the most recent available agricultural production figures (2021/2022 CSA data) instead of the 2016 figures. **Location: Introduction, Page 4, Lines 85,87 & 89.**

**Specific Comment 5:** *Page 5, Line 90 to 91: When you look at the statement started from the later comprised…, some thing is missing.*

**Response:** We rewrote this sentence to ensure complete structural and grammatical clarity. **Location: Materials and Methods, Page 7, Lines 154-155.**

**SECTION 2: RESPONSE TO REVIEWER #3**

**Comment 1:***"Title: The current title appears overly broad. The title should be precise and reflect study specific focused towards the agronomic manipulations. I recommend modifying the title to align with manuscript findings"*

Response: We modified the title to be precise and focused on agronomic manipulations and variety-specific responses. New Title: ' Interactive Effects of Seed Rate, Sowing Method, and Variety on Teff (*Eragrostis tef*) Yield, Yield Components, and Lodging Trait Performance in the Central Highlands of Ethiopia'. Location: Title Page, Line 3-5.

**Comment 2:** *"Abstract: The section is poorly written and needs redrafting, there is lot of ambiguity and delicacy in presenting the values. Since author wants to compare the performance of varieties, but creates confusion while comparing crop with variety. Furthermore, the advantage associated with particular treatment should be quantified."*

**Response:** The Abstract has been completely redrafted. We eliminated ambiguity, provided exact quantified percentage/yield increases for top treatments, and removed unnecessary words as requested. **Location: Abstract, Page 2, Lines 32–46.**

**Comment 3:** *"Line 23 delete the word variety"*

**Response:** We deleted the word 'variety' at Line 23 as instructed. **Location: Abstract, Page 2, Line 35.**

**Comment 4:** *"Line No 25, 26 and 27 are confusing and contradictory."*

**Response:** We rephrased Lines 25–27 to present the treatment comparisons clearly without contradictory statements. **Location: Abstract, Page 2, Lines 40–46.**

**Comment 5:** *"Introduction. The purpose of the research is nicely grounded, however lack of coherence unnecessary lengthening of sentences, overlapping of ideas needs clarity and improvement. Line No 37 redraft this sentence"*

**Response:** We restructured the Introduction, eliminated repetitive sentences, improved logical transitions, and redrafted Line 37 for clarity and conciseness. **Location: Throughout Introduction, Pages 3-6, Line 68-69.**

**Comment 6:** *Material and Methods: Text is clear and provides good details, but needs to be tightened for better academic flow and conciseness.*

**Response:** we have edited the Materials and Methods section to remove wordiness and improve its flow while retaining all technical parameters (design, plot sizes, seed sources, measured traits, and statistical tests). **Location: Throughout the Material and Method Section**

**Comment 7:** *Line No 80: Season should be mentioned.*

**Response:** We have revised the text at Line 80 to explicitly mention the specific growing season during which the field experiment was conducted (2022 main cropping season, Meher**). Location: Materials and Methods, Page 6, Lines 138.**

**Comment 8:** “*Full word should be written at first mention in your text followed by abbreviation in parenthesis.”*

**Response:** We have updated the manuscript text to define all abbreviated terms in full upon their first appearance, followed by the abbreviation in parentheses. **Location: Materials and Methods, Page 9, Lines 163.**

**Comment 9:** *“Results and discussion: While the author mentions significant impact of cultivars on tillering, but the magnitude of its increment/ decrement is lacking in text, which needs further elaboration.”*

**Response:** We extended the tillering discussion by quantifying exact percentage increases/decreases between cultivars. **Location: Results and Discussion, Page 14, Lines 272-277.**

**Comment 10:** *The author should follow the sequence while highlighting the results, like PH should have been discussed first... Under plant height follow proper sequence (main factors first, then interactions)."*

**Response:** We reorganized the Results section sequence: Plant height (PH) is now presented first, followed sequentially by phenological parameters (days to 50% heading), yield components, and finally grain yield and we rearrange the table order. **Location: Throughout the Results and Discussion Section**

**Comment 11:** *"Also the author needs to quantify the advantage associated with the respective treatment."*

**Response:** We have revised the Results section to systematically quantify the advantages of superior treatments explicitly stating percentage increases relative to the control and inferior treatments**. Location: Throughout the Results and Discussion Section**

**Comment 12:**  *"Under the sub heading Plant height, the author shall follow the proper sequence in writing the results, first main factors and then interaction results should be discussed."*

**Response:** We have revised the "Plant Height" and Other parameters to follow a systematic sequence, presenting the main effects of individual factors first, followed by interaction effects, and concluding with the specific quantified advantages of the best treatment combinations. **Location: Results and Discussion, Pages 9, line 188-194, page 17, 331-338, Page 20, 369-375.**

**Comment 13:** *"Under panicle length, author has highlighted the effect of seed rate followed by varieties followed by sowing methods which is contradictory to the table structure as well as same pattern was not followed although eg biomass etc."*

**Response:** We have standardized the sequence of factors across the entire Results section (including text and tables) to ensure complete alignment and logical flow throughout the manuscript. Across all subsections, including Panicle Length and Biomass Yield, Lodging Index the factors and their interactions are now consistently presented in the following order: 1) Variety, 2) Sowing Method, and 3) Seeding Rate. **Location: Throughout the Results and Discussion section: *Panicle Length*, Page 15, Line 301, as well as *Biomass Yield*, page 15, line 340 and associated summary tables (Tables 2-7).**

**Comment 14: *Line 228:*** *Author needs to substantiate the reason for this variable response of genotype.*

**Response*:*** *We have revised the text at Line 228 to explain that the differential performance between cultivars (Quncho vs. Kora) is driven by genotype-by-environment interaction.* **Location: Results and Discussion, Page 16, Lines 316–318.**

**Comment 15:** (Line 281): *Author highlights non-significant effect of varieties on lodging, whereas the values such as Quncho and Kora seem to be significantly different (Table No 6).*

**Response:** The data in Table 6 show a statistically significant difference between cultivars. We **have deleted the erroneous sentence** reflect the significant varietal difference shown in Table 6.

**Comment 16:** *"Author should be more specific rather than optimizing the effects, there is no conclusive finding in the conclusion which highlights remedy to the objectives identified in the introduction. The variety names should be italicized although. I recommend revising the conclusion to clearly link your findings to the productivity concerns.*

**Response:** We have substantially revised the Conclusion section to provide specific, quantifiable recommendations that directly solve the productivity and lodging challenges highlighted in the Introduction. Furthermore, we have ensured all teff variety names (e.g., *Quncho*, *Kora*) are **italicized throughout the entire manuscript**. **Location: *Conclusion*, Page 22-23, Lines 441-449, 456-460.**
