# Supplementary material for: Interactive effects of seed rate, sowing method, and variety on Teff (Eragrostis tef) yield, yield components, and lodging trait performance in the central highlands of Ethiopia
Source: PLoS One. 2026 Oct 12;21(10):e0360297. doi: 10.1371/journal.pone.0360297 (PMC13657313; doi:10.1371/journal.pone.0360297)
Supplement: Supplementary file 4 [file pone.0360297.s007.docx]

I thank the authors for their careful response to my previous comments. All issues raised in my initial review have been adequately addressed. I have no further comments and recommend the manuscript for publication.
