# Supplementary material for: Interactive effects of seed rate, sowing method, and variety on Teff (Eragrostis tef) yield, yield components, and lodging trait performance in the central highlands of Ethiopia
Source: PLoS One. 2026 Oct 12;21(10):e0360297. doi: 10.1371/journal.pone.0360297 (PMC13657313; doi:10.1371/journal.pone.0360297)
Supplement: Peer review history

# Peer review: Decision Letter 0

**DOI:** 10.1371/journal.pone.0360297.r001

**Authors:** Mehdi Rahimi

22 Jul 2026

Dear Dr. Belete,

Thank you for submitting your manuscript to PLOS ONE. After careful consideration, we feel that it has merit but does not fully meet PLOS ONE’s publication criteria as it currently stands. Therefore, we invite you to submit a revised version of the manuscript that addresses the points raised during the review process.

Please submit your revised manuscript by Sep 05 2026 11:59PM. If you will need more time than this to complete your revisions, please reply to this message or contact the journal office at plosone@plos.org. When you're ready to submit your revision, log on to https://www.editorialmanager.com/pone/ and select the 'Submissions Needing Revision' folder to locate your manuscript file.

- A letter that responds to each point raised by the academic editor and reviewer(s). You should upload this letter as a separate file labeled 'Response to Reviewers'.
- A marked-up copy of your manuscript that highlights changes made to the original version. You should upload this as a separate file labeled 'Revised Manuscript with Track Changes'.
- An unmarked version of your revised paper without tracked changes. You should upload this as a separate file labeled 'Manuscript'.

If you would like to make changes to your financial disclosure, please include your updated statement in your cover letter. Guidelines for resubmitting your figure files are available below the reviewer comments at the end of this letter.

If applicable, we recommend that you deposit your laboratory protocols in protocols.io to enhance the reproducibility of your results. Protocols.io assigns your protocol its own identifier (DOI) so that it can be cited independently in the future. For instructions see: https://journals.plos.org/plosone/s/submission-guidelines#loc-laboratory-protocols. Additionally, PLOS ONE offers an option for publishing peer-reviewed Lab Protocol articles, which describe protocols hosted on protocols.io. Read more information on sharing protocols at https://plos.org/protocols?utm_medium=editorial-email&utm_source=authorletters&utm_campaign=protocols.

As the corresponding author, your ORCID iD is verified in the submission system and will appear in the published article. PLOS supports the use of ORCID, and we encourage all coauthors to register for an ORCID iD and use it as well. Please encourage your coauthors to verify their ORCID iD within the submission system before final acceptance, as unverified ORCID iDs will not appear in the published article. *Only*  the individual author can complete the verification step; PLOS staff *cannot*  verify ORCID iDs on behalf of authors.

We look forward to receiving your revised manuscript.

Kind regards,

Mehdi Rahimi, Ph.D.

Academic Editor

PLOS One

Journal Requirements:

When submitting your revision, we need you to address these additional requirements.

1. Please ensure that your manuscript meets PLOS ONE's style requirements, including those for file naming. The PLOS ONE style templates can be found at

https://journals.plos.org/plosone/s/file?id=wjVg/PLOSOne_formatting_sample_main_body.pdf and

https://journals.plos.org/plosone/s/file?id=ba62/PLOSOne_formatting_sample_title_authors_affiliations.pdf

2. In your Methods section, please provide additional information regarding the permits you obtained for the work. Please ensure you have included the full name of the authority that approved the field site access and, if no permits were required, a brief statement explaining why.

3. Please note that PLOS One has specific guidelines on code sharing for submissions in which author-generated code underpins the findings in the manuscript. In these cases, we expect all author-generated code to be made available without restrictions upon publication of the work. Please review our guidelines at https://journals.plos.org/plosone/s/materials-and-software-sharing#loc-sharing-code and ensure that your code is shared in a way that follows best practice and facilitates reproducibility and reuse.

4. Please upload a new copy of Figure 1 as the detail is not clear. Please follow the link for more information:  https://journals.plos.org/plosone/s/figures.

5. We note that your Data Availability Statement is currently as follows: "All relevant data are within the manuscript and its Supporting Information files."

Please confirm at this time whether or not your submission contains all raw data required to replicate the results of your study. Authors must share the “minimal data set” for their submission. PLOS defines the minimal data set to consist of the data required to replicate all study findings reported in the article, as well as related metadata and methods (https://journals.plos.org/plosone/s/data-availability#loc-minimal-data-set-definition).

For example, authors should submit the following data:

- The values behind the means, standard deviations and other measures reported;

- The values used to build graphs;

- The points extracted from images for analysis.

Authors do not need to submit their entire data set if only a portion of the data was used in the reported study.

If your submission does not contain these data, please either upload them as Supporting Information files or deposit them to a stable, public repository and provide us with the relevant URLs, DOIs, or accession numbers. For a list of recommended repositories, please see https://journals.plos.org/plosone/s/recommended-repositories.

If there are ethical or legal restrictions on sharing a de-identified data set, please explain them in detail (e.g., data contain potentially sensitive information, data are owned by a third-party organization, etc.) and who has imposed them (e.g., an ethics committee). Please also provide contact information for a data access committee, ethics committee, or other institutional body to which data requests may be sent. If data are owned by a third party, please indicate how others may request data access.

6. If the reviewer comments include a recommendation to cite specific previously published works, please review and evaluate these publications to determine whether they are relevant and should be cited. There is no requirement to cite these works unless the editor has indicated otherwise.

[Note: HTML markup is below. Please do not edit.]

Reviewers' comments:

Reviewer's Responses to Questions

**Comments to the Author**

1. Is the manuscript technically sound, and do the data support the conclusions?

Reviewer #1: Yes

Reviewer #2: Yes

Reviewer #3: No

**********

2. Has the statistical analysis been performed appropriately and rigorously?

Reviewer #1: Yes

Reviewer #2: Yes

Reviewer #3: N/A

**********

3. Have the authors made all data underlying the findings in their manuscript fully available?

The PLOS Data policy

Reviewer #1: Yes

Reviewer #2: Yes

Reviewer #3: Yes

**********

4. Is the manuscript presented in an intelligible fashion and written in standard English?

Reviewer #1: Yes

Reviewer #2: Yes

Reviewer #3: No

**********

Reviewer #1: Teff (Eragrostis tef) occupies close to three million hectares of agricultural land in

Ethiopia, nonetheless, realized yields remain substantially below the crop’s inherent

genetic yield potential. Thus, this study aimed to examine how different sowing rates

and methods impacted productivity of teff varieties in Mojana Wodera Woreda. The

treatments included three varieties of teff (namely, Quncho, Kora and Abolse (local

variety), three sowing rates (10, 15 and 20 kg ha -1), and three sowing methods (15 cm

and 20 cm spacing between rows and broad casting) . Sowing variety quncho at a rate

of 10 kg ha-1 with a row spacing of 15 cm produced the maximum amount of

aboveground biomass yield (10.76 t ha-1). It also produced the largest panicle (45.14

cm) and yield (2.47 t ha-1). Furthermore, the maximum panicle length (44.46 cm) and

yield (2.32 t ha-1) were recorded when teff was sown 20 cm apart between rows. The

highest grain yield and largest panicle were when teff was seeded at 10 kg ha-1.

Therefore, variety Quncho was the best for the study area, and sowing teff at a rate of

10 kg ha-1 with 20 cm spacing between rows enhanced its productivity.

Reviewer #2: General comments

Generally, the manuscript is written well. However, revision is important to improve the clarity and novelty of the study

Major comments

Abstract

Clearly explain the results

Conclusion

In conclusion, clearly explain the presented result and what it means. Readers expect to see the objective of the study and the key methodology that was not explained.

Minor comments

To the introduction, add the world and continental production status

Explain the source of those two improved varieties under the methodology

Clearly explain the previous finding by including where it was conducted and its gap to increase reliability of this study

This study was only one location and one season, what would be your suggestion for future studies

Specific comments

Page 2, line 22: describe whether it is panicle length or panicle number

Page 2, Line 28: You concluded 10kg per ha rate by 20cm spacing as better productivity for Quncho variety. However, the result you wrote on Line 25 for the same variety Quncho showed better yield and panicle length at 10kg per ha and 15 cm spacing (e.g. 2.47 yield is higher than 2.32 per ha)

Page 2, Line 34: Cite statement with the chromosome number

Page 3, Line 50 to 52: use data of recent cropping season, 2016 is old.

Page 5, Line 90 to 91: When you look at the statement started from the later comprised…, some thing is missing.

Reviewer #3: Title: The current title appears overly broad. The title should be precise and reflect study specific focused towards the agronomic manipulations. I recommend modifying the title to align with manuscript findings

Abstract: The section is poorly written and needs redrafting, there is lot of ambiguity and delicacy in presenting the values. Since author wants to compare the performance of varieties, but creates confusion while comparing crop with variety. Furthermore the advantage associated with particular treatment should be quantified.

Line 23 delete the word variety

Line No 25, 26 and 27 are confusing and contradictory.

Introduction. The purpose of the research is nicely grounded, however lack of coherence unnecessary lengthening of sentences, overlapping of ideas needs clarity and improvement.

Line No 37 redraft this sentence

Material and methods: Text is clear and provides good details, but needs to be tightened for better academic flow and conciseness.

Line No 80 season should be mentioned.

Line No. 89. Treatments should follow similar sequence in tables as well as text and same should be followed throughout.

Line No 96. Full word should be written at first mention in your text followed by abbreviation in parenthesis.

Results and discussion:

Results are clearly written, but author needs to further strengthened this section

While the author mentions significant impact of cultivars on tillering, but the magnitude of its increment/ decrement is lacking in text, which needs further elaboration.

The author should follow the sequence while highlighting the results, like PH should have been discussed first followed by other parameters. Also the author needs to quantify the advantage associated with the respective treatment. Under the sub heading Plant height, the author shall follow the proper sequence in writing the results, first main factors and then interaction results should be discussed.

Under panicle length, author has highlighted the effect of seed rate followed by varieties followed by sowing methods which is contradictory to the table structure as well as same pattern was not followed although eg biomass etc.

Line No.228 author needs to substantiate the reason for this variable response of genotype.

Line No 281: author highlights non-significant effect varieties on lodging, whereas the values such as Qucho and Kora seem to be significantly different (Table No 6),

Conclusion: Author should be more specific rather than optimizing the effects, there is no conclusive finding in the conclusion which highlights remedy to the objectives identified in the introduction. The variety names should be italicized although

I recommend revising the conclusion to clearly link your findings to the productivity concerns.

**********

what does this mean?). If published, this will include your full peer review and any attached files.

If you choose “no”, your identity will remain anonymous but your review may still be made public.

**Do you want your identity to be public for this peer review?**  For information about this choice, including consent withdrawal, please see our Privacy Policy

Reviewer #1: No

Reviewer #2: **Yes:**  Nezif AbaJebal Abadura

Reviewer #3: No

**********

[NOTE: If reviewer comments were submitted as an attachment file, they will be attached to this email and accessible via the submission site. Please log into your account, locate the manuscript record, and check for the action link "View Attachments". If this link does not appear, there are no attachment files.]

To ensure your figures meet our technical requirements, please review our figure guidelines: https://journals.plos.org/plosone/s/figures

You may also use PLOS’s free figure tool, NAAS, to help you prepare publication quality figures: https://journals.plos.org/plosone/s/figures#loc-tools-for-figure-preparation.

NAAS will assess whether your figures meet our technical requirements by comparing each figure against our figure specifications.

Attachment Submitted filename: comments.docx

Attachment Submitted filename: PONE-D-26-15264.pdf

# Author comment: Author response to Decision Letter 1

**DOI:** 10.1371/journal.pone.0360297.r002

3 Sep 2026

Subject: Response to Reviewers for PONE-D-26-15264 Manuscript

Title: “Genotypic Differences in Teff (Eragrostis tef) Yield Response to Sowing Rates and Methods in the Central Highlands of Ethiopia”

Dear Academic Editor,

Thank you very much for giving us the opportunity to revise our manuscript. We also wish to express our sincere gratitude to the reviewers for their constructive feedback and insightful suggestions, which have helped us significantly improve the quality of our manuscript.

Please note that all page and line numbers referenced in our responses below correspond to the marked-up version of the manuscript (Revised Manuscript with Track Changes).

Below, we provide a point-by-point response to each comment raised by the Academic Editor and the reviewers.

Section 1. Response to Academic Editor

Comment 1: Please ensure that your manuscript meets PLOS ONE's style requirements, including those for file naming

Response: We have thoroughly reviewed the journal’s formatting guidelines and updated the manuscript accordingly. All file names, heading structures, table and citation formats have been adjusted to comply with the journal's requirements. Location: Throughout the manuscript

Comment 2: In your Methods section, please provide additional information regarding the permits you obtained for the work.

Response: We have updated the Methods section to state explicitly that no specific permits were required for the site used for this study. Location: Materials and Methods, Page 6-7, Lines 139-142

Comment 3: Please note that PLOS One has specific guidelines on code sharing for submissions in which author-generated code underpins the findings in the manuscript. In these cases, we expect all author-generated code to be made available without restrictions upon publication of the work.

Response: We have complied fully with PLOS ONE’s Data Availability and Code Sharing policies. All underlying raw data and author-generated R code supporting the manuscript’s findings have been made publicly available without restriction to facilitate reproducibility and reuse.

1. Raw Data: The minimal dataset containing all recorded observations for the evaluated traits (plant height, days to heading, number of tillers, panicle length, lodging index, biomass yield, and grain yield) has been provided as Supporting Information (S1 Data).

2. Raw Data: the minimal dataset containing rainfall, maximum and minimum temperature has been provided as Supporting Information (S2 Data)

3. Analysis Code: The full R script used for processing the data, fitting the three-factor ANOVA models, and executing Fisher’s LSD post-hoc mean separations has been provided as Supporting Information (S1 Code).

Comment 4: Please upload a new copy of Figure 1 as the detail is not clear

Response: We apologize for the low resolution of Figure 1 in the previous submission. We have regenerated Figure 1 at high resolution (300 DPI) to ensure all details and text are clear and legible, in accordance with PLOS ONE's figure specifications.

Comment 5: Please confirm at this time whether or not your submission contains all raw data required to replicate the results of your study...

Response: We confirm that our submission contains the complete "minimal data set" required to fully replicate all statistical analyses, means, figures, and study findings reported in the manuscript, without restrictions.

All raw, individual observations behind the reported means and statistical outputs for all evaluated factors (variety, sowing method, and seed rate) and dependent traits (plant height, days to heading, number of tillers, panicle length, lodging index, biomass yield, and grain yield) have been compiled and uploaded as Supporting Information (S1 Data). The graph data points for rainfall, maximum and minimum temperature was uploaded as (S2 Data). Additionally, the R script used to analyze these data is uploaded as (S1 Code).

Comment 6: If the reviewer comments include a recommendation to cite specific previously published works...

Response: We note the journal's policy regarding citation recommendations. None of the reviewers requested or recommended the citation of specific previously published works for this manuscript.

SECTION 2: RESPONSE TO REVIEWER #2

Comment 1: “Abstract: Clearly explain the results”

Response: We have revised the Abstract to clearly present numerical findings, treatment comparisons, and exact percentage/yield advantages rather than generic descriptions. Location: Abstract, Page 2, Lines 32-45.

Comment 2: “Conclusion: In conclusion, clearly explain the presented result and what it means. Readers expect to see the objective of the study and the key methodology that was not explained.”

Response: We have revised the Conclusion section to explicitly state the objective of the study, summarize the key methodology and synthesize the main agronomic findings. Location: Conclusion, Page 22-23, Lines 441-449.

Minor Comment 1: “To the introduction, add the world and continental production status”

Response: We have added recent global and continental teff production status to the Introduction to properly contextualize the crop's global and regional economic importance. Location: Introduction, Page 3, Lines 54-62.

Minor Comment 2: Explain the source of those two improved varieties under the methodology

Response: We added the source for the improved teff varieties under the Materials and Methods section. Location: Materials and Methods, Page 7, Lines 151-154.

Minor Comment 3: Clearly explain the previous finding by including where it was conducted and its gap to increase reliability of this study

Response: We have revised the Introduction section to explicitly detail previous findings, clearly stating the geographic locations and agro-ecological of the studies were conducted. Furthermore, we have highlighted the specific research gaps; regarding variety, seed rate and sowing method interactions. Location: Introduction, Pages 5–6, Lines 109-126.

Minor Comment 4: This study was only one location and one season, what would be your suggestion for future studies

Response: We explicitly acknowledged this single-location, single-season study limitation in the Conclusion sections, adding a recommendation for multi-location and multi-year trials across contrasting agro-ecological zones. Location: Conclusion, Pages 23, Lines 456-460.

Specific Comment 1: Page 2, line 22: describe whether it is panicle length or panicle number

Response: We confirm that the measured parameter is panicle length (cm)

Specific Comment 2 : Page 2, Line 28: You concluded 10kg per ha rate by 20cm spacing as better productivity for Quncho variety. However, the result you wrote on Line 25 for the same variety Quncho showed better yield and panicle length at 10kg per ha and 15 cm spacing (e.g. 2.47 yield is higher than 2.32 per ha)"

Response: We have clarified the reporting of our statistical results to address this area of potential confusion. The value of 2.32 t ha⁻¹ represents the main effect for the sowing method, whereas 2.47 t ha⁻¹ represents the yield for the Quncho variety. We have updated the text on Page 2 to explicitly distinguish treatment means. Location: Abstract, Pages 2, Lines 43–46.

Specific Comment 3: Page 2, Line 34: Cite statement with the chromosome number

Response: We added the official citation supporting the chromosome number of teff (Eragrostis tef,) at this statement. Location: Introduction, Page 3, Line 66.

Specific Comment 4: Page 3, Line 50 to 52: use data of recent cropping season, 2016 is old

Response: We updated the statistical data to reflect the most recent available agricultural production figures (2021/2022 CSA data) instead of the 2016 figures. Location: Introduction, Page 4, Lines 85,87 & 89.

Specific Comment 5: Page 5, Line 90 to 91: When you look at the statement started from the later comprised…, some thing is missing.

Response: We rewrote this sentence to ensure complete structural and grammatical clarity. Location: Materials and Methods, Page 7, Lines 154-155.

SECTION 2: RESPONSE TO REVIEWER #3

Comment 1:"Title: The current title appears overly broad. The title should be precise and reflect study specific focused towards the agronomic manipulations. I recommend modifying the title to align with manuscript findings"

Response: We modified the title to be precise and focused on agronomic manipulations and variety-specific responses. New Title: ' Interactive Effects of Seed Rate, Sowing Method, and Variety on Teff (Eragrostis tef) Yield, Yield Components, and Lodging Trait Performance in the Central Highlands of Ethiopia'. Location: Title Page, Line 3-5.

Comment 2: "Abstract: The section is poorly written and needs redrafting, there is lot of ambiguity and delicacy in presenting the values. Since author wants to compare the performance of varieties, but creates confusion while comparing crop with variety. Furthermore, the advantage associated with particular treatment should be quantified."

Response: The Abstract has been completely redrafted. We eliminated ambiguity, provided exact quantified percentage/yield increases for top treatments, and removed unnecessary words as requested. Location: Abstract, Page 2, Lines 32–46.

Comment 3: "Line 23 delete the word variety"

Response: We deleted the word 'variety' at Line 23 as instructed. Location: Abstract, Page 2, Line 35.

Comment 4: "Line No 25, 26 and 27 are confusing and contradictory."

Response: We rephrased Lines 25–27 to present the treatment comparisons clearly without contradictory statements. Location: Abstract, Page 2, Lines 40–46.

Comment 5: "Introduction. The purpose of the research is nicely grounded, however lack of coherence unnecessary lengthening of sentences, overlapping of ideas needs clarity and improvement. Line No 37 redraft this sentence"

Response: We restructured the Introduction, eliminated repetitive sentences, improved logical transitions, and redrafted Line 37 for clarity and conciseness. Location: Throughout Introduction, Pages 3-6, Line 68-69.

Comment 6: Material and Methods: Text is clear and provides good details, but needs to be tightened for better academic flow and conciseness.

Response: we have edited the Materials and Methods section to remove wordiness and improve its flow while retaining all technical parameters (design, plot sizes, seed sources, measured traits, and statistical tests). Location: Throughout the Material and Method Section

Comment 7: Line No 80: Season should be mentioned.

Response: We have revised the text at Line 80 to explicitly mention the specific growing season during which the field experiment was conducted (2022 main cropping season, Meher). Location: Materials and Methods, Page 6, Lines 138.

Comment 8: “Full word should be written at first mention in your text followed by abbreviation in parenthesis.”

Response: We have updated the manuscript text to define all abbreviated terms in full upon their first appearance, followed by the abbreviation in parentheses. Location: Materials and Methods, Page 9, Lines 163.

Comment 9: “Results and discussion: While the author mentions significant impact of cultivars on tillering, but the magnitude of its increment/ decrement is lacking in text, which needs further elaboration.”

Response: We extended the tillering discussion by quantifying exact percentage increases/decreases between cultivars. Location: Results and Discussion, Page 14, Lines 272-277.

Comment 10: The author should follow the sequence while highlighting the results, like PH should have been discussed first... Under plant height follow proper sequence (main factors first, then interactions)."

Response: We reorganized the Results section sequence: Plant height (PH) is now presented first, followed sequentially by phenological parameters (days to 50% heading), yield components, and finally grain yield and we rearrange the table order. Location: Throughout the Results and Discussion Section

Comment 11: "Also the author needs to quantify the advantage associated with the respective treatment."

Response: We have revised the Results section to systematically quantify the advantages of superior treatments explicitly stating percentage increases relative to the control and inferior treatments. Location: Throughout the Results and Discussion Section

Comment 12: "Under the sub heading Plant height, the author shall follow the proper sequence in writing the results, first main factors and then interaction results should be discussed."

Response: We have revised the "Plant Height" and Other parameters to follow a systematic sequence, presenting the main effects of individual factors first, followed by interaction effects, and concluding with the specific quantified advantages of the best treatment combinations. Location: Results and Discussion, Pages 9, line 188-194, page 17, 331-338, Page 20, 369-375.

Comment 13: "Under panicle length, author has highlighted the effect of seed rate followed by varieties followed by sowing methods which is contradictory to the table structure as well as same pattern was not followed although eg biomass etc."

Response: We have standardized the sequence of factors across the entire Results section (including text and tables) to ensure complete alignment and logical flow throughout the manuscript. Across all subsections, including Panicle Length and Biomass Yield, Lodging Index the factors and their interactions are now consistently presented in the following order: 1) Variety, 2) Sowing Method, and 3) Seeding Rate. Location: Throughout the Results and Discussion section: Panicle Length, Page 15, Line 301, as well as Biomass Yield, page 15, line 340 and associated summary tables (Tables 2-7).

Comment 14: Line 228: Author needs to substantiate the reason for this variable response of genotype.

Response: We have revised the text at Line 228 to explain that the differential performance between cultivars (Quncho vs. Kora) is driven by genotype-by-environment interaction. Location: Results and Discussion, Page 16, Lines 316–318.

Comment 15: (Line 281): Author highlights non-significant effect of varieties on lodging, whereas the values such as Quncho and Kora seem to be significantly different (Table No 6).

Response: The data in Table 6 show a statistically significant difference between cultivars. We have deleted the erroneous sentence reflect the significant varietal difference shown in Table 6.

Comment 16: "Author should be more specific rather than optimizing the effects, there is no conclusive finding in the conclusion which highlights remedy to the objectives identified in the introduction. The variety names should be italicized although. I recommend revising the conclusion to clearly link your findings to the productivity concerns.

Response: We have substantially revised the Conclusion section to provide specific, quantifiable recommendations that directly solve the productivity and lodging challenges highlighted in the Introduction. Furthermore, we have ensured all teff variety names (e.g., Quncho, Kora) are italicized throughout the entire manuscript. Location: Conclusion, Page 22-23, Lines 441-449, 456-460.

Attachment Submitted filename: Response to Reviewers .docx

# Peer review: Decision Letter 1

**DOI:** 10.1371/journal.pone.0360297.r003

**Authors:** Mehdi Rahimi

24 Sep 2026

Title:  Interactive Effects of Seed Rate, Sowing Method, and Variety on Teff (Eragrostis tef) Yield, Yield Components, and Lodging Trait Performance in the Central Highlands of Ethiopia

PONE-D-26-15264R1

Dear Dr. Belete,

We’re pleased to inform you that your manuscript has been judged scientifically suitable for publication and will be formally accepted for publication once it meets all outstanding technical requirements.

Within one week, you’ll receive an e-mail detailing the required amendments. When these have been addressed, you’ll receive a formal acceptance letter and your manuscript will be scheduled for publication.

An invoice will be generated when your article is formally accepted. Please note, if your institution has a publishing partnership with PLOS and your article meets the relevant criteria, all or part of your publication costs will be covered. Please make sure your user information is up-to-date by logging into Editorial Manager at Editorial Manager® and clicking the ‘Update My Information' link at the top of the page. For questions related to billing, please contact billing support.

If your institution or institutions have a press office, please notify them about your upcoming paper to help maximize its impact. If they’ll be preparing press materials, please inform our press team as soon as possible -- no later than 48 hours after receiving the formal acceptance. Your manuscript will remain under strict press embargo until 2 pm Eastern Time on the date of publication. For more information, please contact onepress@plos.org.

Kind regards,

Mehdi Rahimi, Ph.D.

Academic Editor

PLOS One

Additional Editor Comments (optional):

Reviewers' comments:

Reviewer's Responses to Questions

**Comments to the Author**

Reviewer #2: All comments have been addressed

**********

2. Is the manuscript technically sound, and do the data support the conclusions?

Reviewer #2: Yes

**********

3. Has the statistical analysis been performed appropriately and rigorously?

Reviewer #2: Yes

**********

4. Have the authors made all data underlying the findings in their manuscript fully available?

The PLOS Data policy

Reviewer #2: Yes

**********

5. Is the manuscript presented in an intelligible fashion and written in standard English?

Reviewer #2: Yes

**********

Reviewer #2: I thank the authors for their careful response to my previous comments. All issues raised in my initial review have been adequately addressed. I have no further comments and recommend the manuscript for publication.

**********

what does this mean?). If published, this will include your full peer review and any attached files.

If you choose “no”, your identity will remain anonymous but your review may still be made public.

**Do you want your identity to be public for this peer review?**  For information about this choice, including consent withdrawal, please see our Privacy Policy

Reviewer #2: **Yes:**  Nezif Abajebal Abadura

**********

Attachment Submitted filename: attachment.docx

# Editor report: Acceptance letter

**DOI:** 10.1371/journal.pone.0360297.r004

**Authors:** Mehdi Rahimi

PONE-D-26-15264R1

PLOS One

Dear Dr. Belete,

I'm pleased to inform you that your manuscript has been deemed suitable for publication in PLOS One. Congratulations! Your manuscript is now being handed over to our production team.

At this stage, our production department will prepare your paper for publication. This includes ensuring the following:

* All references, tables, and figures are properly cited

* All relevant supporting information is included in the manuscript submission,

* There are no issues that prevent the paper from being properly typeset

You will receive further instructions from the production team, including instructions on how to review your proof when it is ready. Please keep in mind that we are working through a large volume of accepted articles, so please give us a few days to review your paper and let you know the next and final steps.

Lastly, if your institution or institutions have a press office, please let them know about your upcoming paper now to help maximize its impact. If they'll be preparing press materials, please inform our press team within the next 48 hours. Your manuscript will remain under strict press embargo until 2 pm Eastern Time on the date of publication. For more information, please contact onepress@plos.org.

You will receive an invoice from PLOS for your publication fee after your manuscript has reached the completed accept phase. If you receive an email requesting payment before acceptance or for any other service, this may be a phishing scheme. Learn how to identify phishing emails and protect your accounts at https://explore.plos.org/phishing.

If we can help with anything else, please email us at customercare@plos.org.

Thank you for submitting your work to PLOS One and supporting open access.

Kind regards,

PLOS One Editorial Office Staff

on behalf of

Associate Prof. Mehdi Rahimi

Academic Editor

PLOS One
